# Systematic, multiparametric analysis of *Mycobacterium tuberculosis* intracellular infection offers insight into coordinated virulence

**DOI:** 10.1371/journal.ppat.1006363

**Published:** 2017-05-15

**Authors:** Amy K. Barczak, Roi Avraham, Shantanu Singh, Samantha S. Luo, Wei Ran Zhang, Mark-Anthony Bray, Amelia E. Hinman, Matthew Thompson, Raymond M. Nietupski, Aaron Golas, Paul Montgomery, Michael Fitzgerald, Roger S. Smith, Dylan W. White, Anna D. Tischler, Anne E. Carpenter, Deborah T. Hung

**Affiliations:** 1 Division of Infectious Diseases, Massachusetts General Hospital, Boston, Massachusetts, United States of America; 2 The Ragon Institute of Harvard, MIT, and Massachusetts General Hospital, Cambridge, Massachusetts, United States of America; 3 Department of Medicine, Harvard Medical School, Boston, Massachusetts, United States of America; 4 The Broad Institute, Cambridge, Massachusetts, United States of America; 5 Department of Molecular Biology and Center for Computational and Integrative Biology, Massachusetts General Hospital, Boston, Massachusetts, United States of America; 6 Department of Microbiology and Immunology and Center for Infectious Diseases and Microbiology Translational Research, University of Minnesota Twin Cities, Minneapolis, Minnesota, United States of America; 7 Department of Genetics, Harvard Medical School, Boston, Massachusetts, United States of America; National Institutes of Health, UNITED STATES

## Abstract

A key to the pathogenic success of *Mycobacterium tuberculosis* (*Mtb*), the causative agent of tuberculosis, is the capacity to survive within host macrophages. Although several factors required for this survival have been identified, a comprehensive knowledge of such factors and how they work together to manipulate the host environment to benefit bacterial survival are not well understood. To systematically identify *Mtb* factors required for intracellular growth, we screened an arrayed, non-redundant *Mtb* transposon mutant library by high-content imaging to characterize the mutant-macrophage interaction. Based on a combination of imaging features, we identified mutants impaired for intracellular survival. We then characterized the phenotype of infection with each mutant by profiling the induced macrophage cytokine response. Taking a systems-level approach to understanding the biology of identified mutants, we performed a multiparametric analysis combining pathogen and host phenotypes to predict functional relationships between mutants based on clustering. Strikingly, mutants defective in two well-known virulence factors, the ESX-1 protein secretion system and the virulence lipid phthiocerol dimycocerosate (PDIM), clustered together. Building upon the shared phenotype of loss of the macrophage type I interferon (IFN) response to infection, we found that PDIM production and export are required for coordinated secretion of ESX-1-substrates, for phagosomal permeabilization, and for downstream induction of the type I IFN response. Multiparametric clustering also identified two novel genes that are required for PDIM production and induction of the type I IFN response. Thus, multiparametric analysis combining host and pathogen infection phenotypes can be used to identify novel functional relationships between genes that play a role in infection.

## Author summary

Tuberculosis (TB) remains a significant global health problem. One barrier to developing novel approaches to preventing and treating TB is an incomplete understanding of the strategies that the causative bacterium, *Mycobacterium tuberculosis* (*Mtb*), uses to survive and cause disease in the host. To systematically identify *Mtb* genes required for growth in infected host cells, we screened an annotated, arrayed library of *Mtb* mutants in macrophages using high-content imaging. We then used multiplexed cytokine analysis to profile the macrophage response to each mutant attenuated for intracellular growth. Combining imaging parameters reflective of intracellular infection with the macrophage response to each mutant, we predicted novel functional relationships between *Mtb* genes required for infection. We then validated these predictions by demonstrating that production and export of a cell envelope lipid is required for coordinated virulence-associated protein secretion, phagosomal membrane rupture, and production of the macrophage type I interferon response. Extending our prediction of functional relationships to unknown genes, we demonstrated that two genes not previously linked to virulence also act in this pathway. This work demonstrates a broadly applicable approach to elucidating and relating bacterial functions required for pathogenesis and demonstrates a previously unknown dependence of *Mtb* virulence-associated protein secretion on an outer envelope lipid.

## Introduction

A key to the pathogenic success of *Mycobacterium tuberculosis* (*Mtb*), the causative agent of tuberculosis (TB), is the capacity to survive within host macrophages. Although several *Mtb* factors required for this survival have been identified [1], a comprehensive knowledge of such factors and how they work together to evade host clearance mechanisms remains elusive. Because cellular models of *Mtb* infection are tractable for high-throughput applications and facilitate mechanistic follow-up studies, these models are useful for identifying and understanding *Mtb* factors that drive the outcome of infection at the host-pathogen interface.

Several screening approaches have identified *Mtb* factors required for its intracellular survival. One approach has focused on early cellular events, specifically *Mtb*’s inhibition of phagosome maturation and acidification. Using large mixed pools of *Mtb* mutants, Pethe *et al* identified mutants that co-localized with iron-containing lysosomes [2]. Similarly, using high-content imaging to screen a library of 11,000 randomly selected, arrayed transposon mutants, Brodin *et al* identified 10 mutants unable to block phagosome maturation based on co-localization with LysoTracker-stained acidified lysosomes [3]. An alternative genomic approach focused on *Mtb* growth in macrophages and compared input and output pools of transposon mutants to identify several attenuated mutants [4]. Interestingly, there is little overlap between the sets of *Mtb* genes identified as important in macrophages using each of these approaches. Of the 10 genes identified by Brodin et al., only one (*pstS3*) was identified in the screen by Rengarajan et al., and only one (*fadD28*) was identified by Pethe et al. There is no overlap between genes identified by Rengarajan et al. and those identified by Pethe et al.

To more systematically identify *Mtb* factors required for intracellular growth, we screened a 2660 member arrayed, non-redundant *Mtb* (H37Rv strain) transposon mutant library by high-content imaging to characterize the mutant-macrophage interaction. Taking a systems-level approach to understanding how the genes disrupted in these mutants might work, we profiled host cytokine secretion in response to infection with each of the 361 mutants most impaired for intracellular survival based on the imaging assay. Subsets of these mutants induced strikingly different host responses, suggesting that the disrupted genes play distinct roles in infection. Using a guilt-by-association approach, we combined clustering by the distinct infection phenotypes of imaging outcome and induced macrophage cytokine response, and identified several groups of *Mtb* mutants predicted to be functionally related. Strikingly, mutants defective in two well-known virulence factors, the ESX-1 protein secretion system and the virulence lipid phthiocerol dimycocerosate (PDIM), clustered together, suggesting a potential functional relationship.

ESX-1 type VII protein secretion has long been known to be a critical virulence function of *Mtb* in macrophages [5] [6]. ESX-1 has been proposed to permeabilize the *Mtb*-containing phagosome, thereby facilitating the transport of *Mtb* genomic DNA into the cytosol and its subsequent detection by the cytosolic surveillance program (CSP) [7]. CSP detection of bacterial DNA through the pathogen recognition receptor cGAS then triggers induction of the macrophage type I interferon (IFN) response [8] [9,10]. Until very recently, ESX-1 was the only bacterial function described as required for phagosomal permeabilization and the subsequent induction of the macrophage type I IFN response.

The cell-surface lipid PDIM has long been linked to virulence as well [11,12], although the mechanism has not been fully determined. Situated predominantly in the cell envelope, PDIM has been proposed to alter membrane properties important for uptake by macrophages [13] and to mask recognition of the bacterial cell by pathogen-recognition receptors (PRRs) in a MYD88-dependent fashion [14]. Here, we find that ESX-1 mutants and PDIM synthesis and export mutants share a common multiparametric phenotype of infection. While it is not surprising that they share similar characteristics of attenuation within infected macrophages, remarkably, they also both failed to induce the macrophage type I IFN response upon infection—a phenotype previously attributed solely to ESX-1 mutants. Based on this shared phenotype, we hypothesized a functional relationship between ESX-1 and PDIM. Indeed, we demonstrate that PDIM production and export are required for the coordinated secretion of ESX-1 substrates. We show that PDIM is required for secretion of ESAT-6, which has previously been implicated in phagosomal rupture [15,16], but is not mandatory for secretion of its presumed obligate heterodimeric partner, CFP-10. Of the other two *Mtb* type VII secretion systems associated with virulence, PDIM is similarly required for secretion of ESX-5 substrates PPE41 and EsxN, but is not required for secretion of the ESX-3 substrates EsxG and EsxH, suggesting a relatively specific relationship between PDIM and secretion of individual substrates.

Extending our prediction of functional relationships based on multiparametric clustering, we tested several mutants of genes of unknown function that also clustered together with PDIM and ESX-1 to determine whether they impact PDIM production or export, ESX-1-mediated secretion, or were required for the type I IFN response through an independent, complementary mechanism. Indeed, we found two mutants (*Rv0712* and *hrp1* (*Rv2626c*)) that fail to induce the type I IFN response because of a defect in PDIM production, thereby assigning novel roles in pathogenesis to these two respective genes. Thus, multiparametric clustering of infection combining detailed bacterial phenotype and host response to *Mtb* mutants can allow a guilt-by-association analysis to identify novel functional relationships between genes that play a role in infection.

## Results

### High-content imaging screen of an arrayed *M. tuberculosis* transposon mutant library for growth in macrophages

To perform high-throughput monitoring of intracellular growth of a library of *Mtb* transposon mutants (S1 Table), we significantly modified our previously developed high-content imaging assay designed for high-throughput chemical screening [17] in order to accommodate heterogeneous bacterial growth among arrayed mutants and the need for external bacterial fluorescent staining by auramine-rhodamine for visualization (full details of assay and imaging pipeline development in S1 Text). Using rifampin in dose response to inhibit *Mtb* growth in macrophages, we developed a training set of images that represented a range of intracellular growth inhibition as defined by colony forming units (CFU). Based on correlation with CFU, we selected three readily-interpretable features as imaging metrics: percent of total macrophages infected with *Mtb* (“Percent infected”), normalized and integrated bacterial fluorescence intensity (“*Mtb* FI”), and macrophage cell count (“Macrophage count”) (S1A Fig). Of note, very similar metrics were used in a previous high-content imaging assay of *Mtb* growth in macrophages applied to small molecule screening [18]. A pilot screen of 190 mutants was then performed to assess assay performance (S1B Fig). The pilot screen data was additionally used to determine a linear combination of all three imaging metrics that would best identify true positives as determined by CFU retesting of predicted hits in comparison with wild-type control. The first principal component (PC1) from a principal component analysis computed on the data was observed to outperform all other metrics (S1C and S1D Fig).

**Fig 1:**
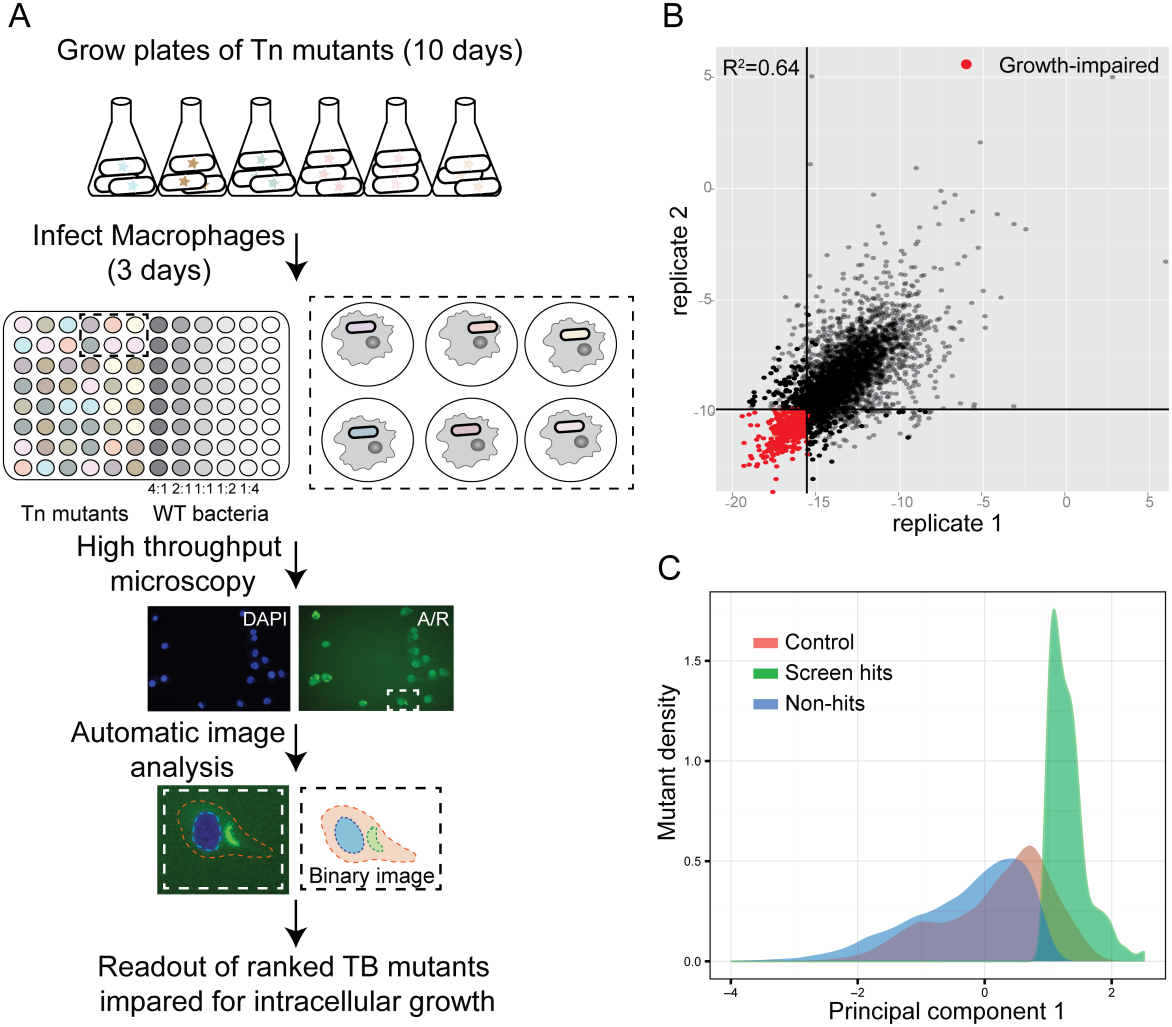
Identification of *M*. *tuberculosis* mutants defective for intracellular growth using a high-content imaging screen. (A) Protocol for the high-content imaging assay for the transposon mutant library growth in macrophages. Wild-type *Mtb* at MOIs varying two-fold from 2:1 to 0.25:1 was included in each plate to allow both accurate normalization between plates and validation of a cutoff for hits based on control population behavior. Because the transposon mutant library does not express a fluorescent protein, *Mtb* was externally stained with auramine-rhodamine after fixation to permit visualization. (B) Correlation of PC1 score of technical replicates in the full screen. (C) Density function of PC1 values for mutants and controls. While hits were selected based on comparison with OD-matched mutants, the distribution of hit mutants falls almost entirely below the wild-type control distribution as well, confirming that hit mutants also behave differently than wild-type.

Using our optimized assay and analysis (Fig 1A), we then screened the remainder of the arrayed, non-redundant *Mtb* 2660 transposon mutant library (Fig 1B, S1E and S2 Figs). Because *Mtb* mutant growth in macrophages is a graded rather than binary phenotype, we defined a threshold for calling hits for targeted follow-up studies. For each mutant, we calculated a weighted average of the 3 imaging outputs (PC1), and the 361 mutants (13.4% of the mutants screened) with the lowest PC1 scores were selected for additional study as our defined set of growth-impaired mutants (S2 Table, Fig 1C). In addition to 15 mutants identified in our screen that had already been confirmed to be growth-impaired in macrophages in the literature, we retested 73 of the top mutants by enumerating surviving bacteria by plating for CFU in comparison with wild-type *Mtb*. Although the biology captured by CFU measurements is limited in comparison with complex imaging phenotypes, CFU is considered the gold-standard measurement of comparative bacterial growth, and was thus used as a metric by which our imaging output was optimized and against which we tested hits. Combining the 47 CFU-confirmed and 15 literature-confirmed hits, a true positive rate of 72% was obtained for identifying mutants defective for intracellular growth (S3 Table). Several hits from previous screens [2–4] were among the identified mutants, thereby providing biological validation of our results (S2 Table).

Several of the identified mutants fit well into the broader context of pathways and functions known to be important for *Mtb* survival in the host (S4 Table). Our screen identified genes in the ESX-1 type VII protein secretion system, perhaps the best-characterized mycobacterial virulence factor [5–7,19,20]. We identified mutants in loci that produce known virulence lipids, including PDIM [11,12]. We additionally identified multiple PE and PPE family genes [21], genes required for synthesis of molybdopterin cofactor [22], the two-component regulator *senX3* [23], and regulators of *Mtb* metal content [24]. Host cholesterol is an important carbon source for intracellular *Mtb* [25,26]; we identified multiple enzymes in the cholesterol catabolic pathway. Finally, we identified several genes involved in nitrogen acquisition or metabolism, supporting the recent recognition of the importance of *Mtb* nitrogen metabolism in infection [27].

### Multiparametric analysis of mutants impaired for intracellular growth identifies clusters of mutants predicted to be functionally related

A more comprehensive knowledge of the *Mtb* factors required for growth in macrophages is an important step in identifying the bacterium’s strategies for success. However, understanding the contribution of each gene to overall virulence and induced host responses as well as the networks that coordinate their functions would offer significant breadth and depth to our current understanding of the complex dynamic between pathogen and host. We thus sought to characterize the mutants identified in the screen both by the phenotype of bacterial growth restriction and by elicited host response. By combining these orthogonal phenotypes in a multiparametric analysis, we hoped to identify, through unbiased cluster analysis, genes that might be functionally related or act in a coordinated manner during infection.

While mutants of interest were initially selected based on an analysis pipeline trained to identify mutants with decreased CFU, in fact the biology of mutants captured in images is richer and more complex than a simple CFU measurement. To fully capitalize on the phenotypic complexity of bacterial growth restriction afforded by imaging, we sought to analyze the mutants based on individual imaging metrics that allow us to distinguish different mutants and the roles of the corresponding genes in infection. We ranked all screened mutants into deciles by score for each of the three individual imaging metrics used in the primary screen PCA and then grouped the mutants based on the similarities of their scores across all three. The majority of hits behaved as one might intuitively expect: higher macrophage survival, lower percent macrophages infected, and lower bacterial fluorescence intensity than the vast majority of screened mutants. However, some mutants behaved differently, eliciting more macrophage cell death or higher percent macrophage infection than the majority of mutants.

**Fig 2:**
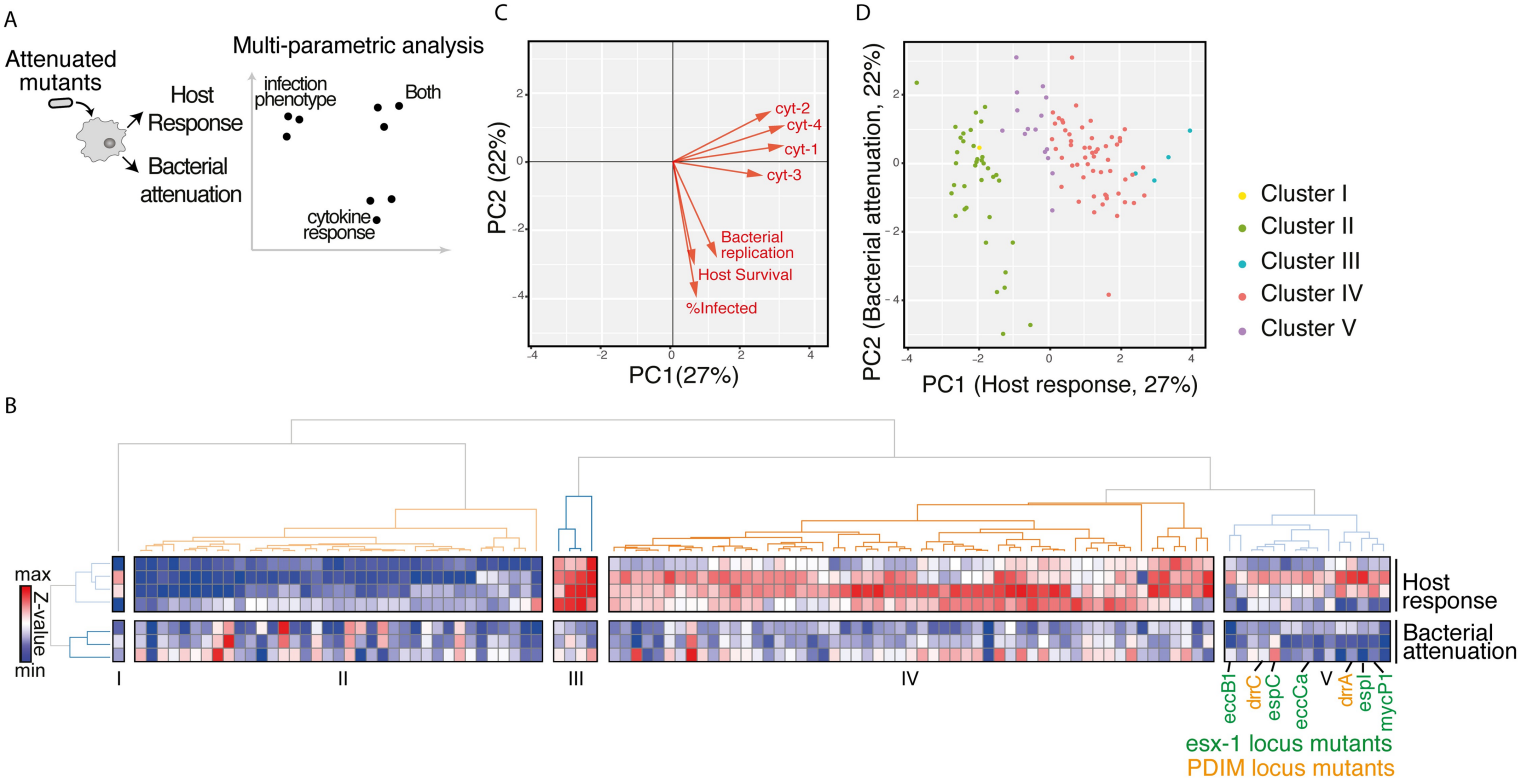
Multiparametric analysis of mutants impaired for intracellular growth identified subsets with shared complex phenotypes. (A) Schematic diagram of the approach to analyzing the mutants. Bacterial attenuation was analyzed based on three imaging parameters (*Mtb* fluorescence intensity, percent infected, and macrophage count) and host response was analyzed based on multiplexed cytokine analysis for each mutant. The phenotypes for each mutant were then combined into a multiparametric analysis as described in B. (B) Unsupervised 2-D clustering of mutants based on a multiparametric representation of their phenotypes. This analysis combines the four host response clusters (top 4 rows) and three bacterial imaging clusters (bottom 3 rows). Each column represents one of the 113 mutants included in the analysis. Colors indicate low (blue) to high (red) normalized values for each phenotype. Mutants cluster into five groups (Clusters I-V). Mutants in the PDIM locus (labels in orange) and ESX-1 locus (labels in green) group together in Cluster V. (C) Contribution of each bacterial imaging phenotype (bacterial replication, host survival, and % infected: orange vectors) and clustered cytokine response phenotype (cyt-1, cyt-2, cyt-3, and cyt-4: black vectors) to principal component 1 (PC1) and principal component 2 (PC2) of the principal component analysis. PC1 is comprised almost entirely of cytokine phenotypes while PC2 is comprised almost entirely of bacterial imaging features, suggesting that the two sets of features represent orthogonal and complementary information. (D) The 113 mutants included in the multiparametric analysis separate when plotted by PC1 and PC2. Included mutants were those that induced macrophage responses most distinct from the response to wild-type *Mtb*. Coloring of mutant clusters reflects mapping of the analysis performed in 2D back to the PC1/PC2 graph.

To characterize the host response elicited by each mutant, we then used a multiplexed Luminex assay to quantify macrophage production of cytokines in response to infection with each of the 361 mutants identified in the primary screen. Twelve cytokines were accurately quantifiable and differed significantly between wild-type-infected and subsets of mutant-infected cells (S3 Fig). These cytokines reflect processes known to be important for *Mtb* macrophage infection, including the type I IFN response (*e*.*g*. CCL5) and pro-inflammatory response (e.g. MMP9, TNF-α). As reflected in these 12 cytokines, macrophage responses to infection with the mutants were in fact quite varied (S4 Fig); this variability did not reflect either the input ratio of bacteria to macrophage or relative degree of intracellular growth impairment (S4B Fig), suggesting that the cytokine response to each mutant is independent of relative attenuation in macrophages. We hypothesized that combining imaging and cytokine phenotypes for each mutant would yield the most information about how those mutants might work together in networks interacting with aspects of the host response to infection (Fig 2A).

To cluster mutants based on both the imaging phenotype, which we considered to reflect primarily bacterial outcomes, and cytokine phenotype, which we considered to reflect primarily host response, we thus sought to combine the two phenotypes in a multiplexed analysis. For this clustering analysis, we had to explicitly balance the contribution of the imaging and cytokine features to avoid either source dominating the analysis. A straightforward approach to doing this is to select the same or similar number of measurements from both sources. To reduce the number of cytokine features, we grouped the features using hierarchical clustering and found that they fell into four groups (cyt-1 (CXCL10 and CCL5), cyt-2 (TNF-α, CXCL2, MMP9, and Lipocalin), cyt-3 (CCL2, CCL4, IGF-1, and BAFF), and cyt-4 (CCL3 and osteopontin) S4C and S4D Fig). Validating the biological relevance of these groupings, cytokines reflecting the type I IFN response clustered together, and pro-inflammatory cytokines clustered together in a distinct group. The features in each group were combined by averaging; we used this grouping for further analysis.

To maximize our ability to distinguish phenotypically unique sets of mutants, we limited analysis from this point to the 113 mutants impaired for intracellular growth with macrophage responses most distinct from wild-type infected cells. Of note, confirmed true positives (by CFU) were represented in both this set of 113 mutants and mutants that induced macrophages responses more similar to wild-type *Mtb*, supporting the idea that the induced macrophage response is a distinct phenotypic readout. Given that the bacterial imaging and host response phenotypes provide distinct information about the mutants, we hypothesized that mutants similar in both metrics would be the most closely functionally related. To identify such phenotypically similar groups of mutants, we then performed a two dimensional unsupervised clustering based on the combined host response features and bacterial imaging features (Fig 2B). This clustering identified five groups of mutants (S5 Table).

To determine objectively whether cytokine and imaging features contributed overlapping or distinct information to the analysis of each mutant, we next performed a principal component analysis to determine how features were related to one another. Interestingly, the first principal component (PC1) was comprised almost entirely of host response features (cyt-1, cyt-2, cyt-3, and cyt-4), while the second principal component (PC2) was comprised almost entirely of bacterial imaging phenotype features (Fig 2C). These results suggest that the bacterial imaging phenotypes and host response phenotypes are in fact nearly orthogonal, and provide complementary information about the tested mutants. Plotting the mutants by PC1 and PC2, we then determined that the mutants indeed segregate in two-dimensional space, indicating that the multiparametric analysis can provide insights into points of divergence in the host-pathogen interaction (Fig 2D). Given the unique phenotypic signature of each cluster in this combined analysis, we hypothesized the mutants within each cluster may be functionally related.

### Synthesis and export of virulence lipid PDIM is required for induction of the macrophage type I IFN response

**Fig 3:**
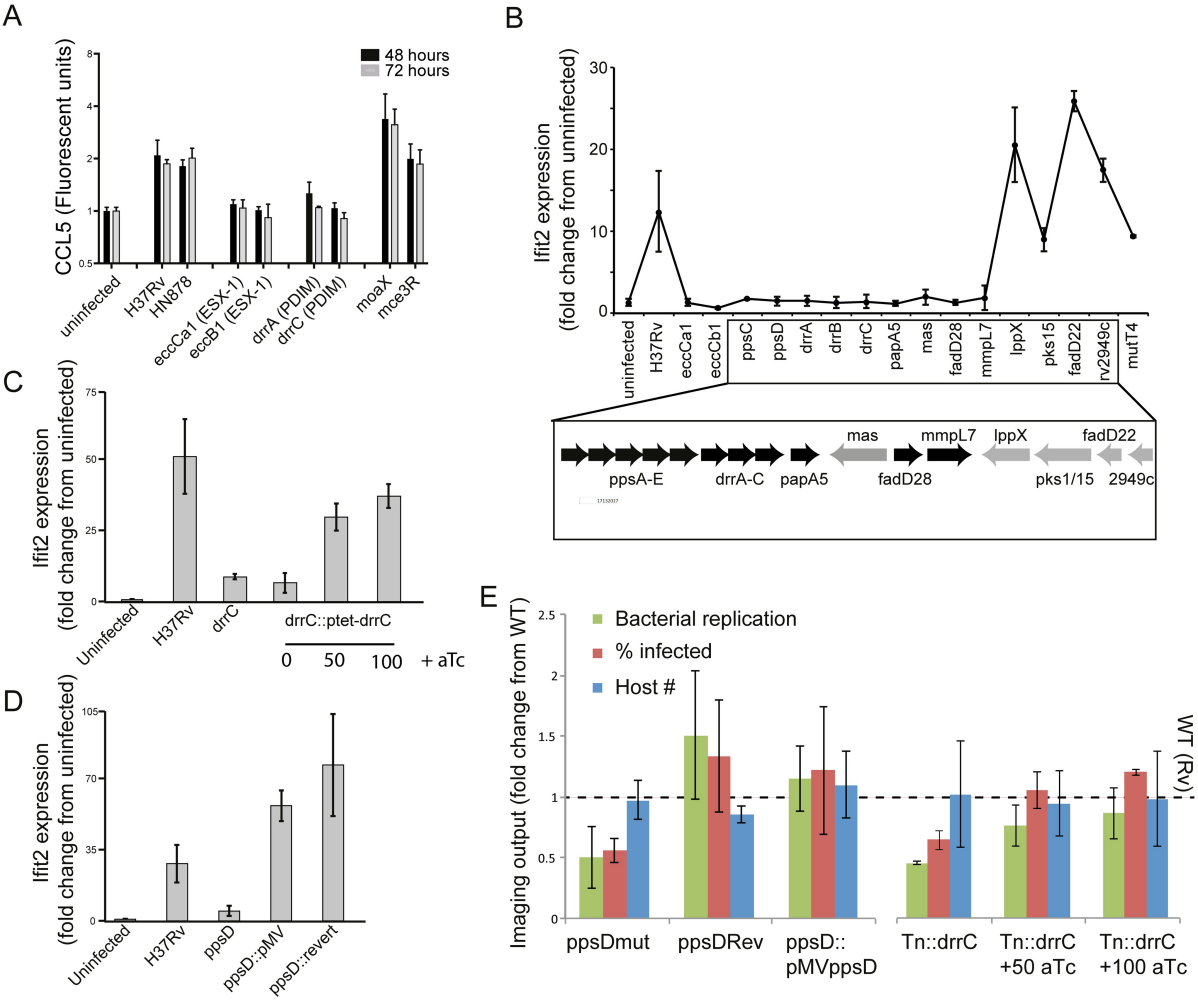
PDIM production and export is required for induction of the macrophage type I IFN response. (A) Measurement of macrophage CCL5 production after infection with *Mtb* strains. J774.A1 cells were infected with wild-type *Mtb* strains or transposon mutants at an MOI of 1:1. Supernatants were harvested at 48 or 72 hours after infection for cytokine quantitation using Luminex. Mean +/- SD of three biological replicates. (B-D) The type I IFN response was measured by quantitation of expression of the type I IFN-responsive gene *ifit2* expression after macrophage infection with *Mtb*. Bone marrow-derived macrophages were infected with the indicated *Mtb* strains at an MOI of 2:1 and RNA was harvested at 24 hours for gene expression analysis by qRT-PCR. (B) Transposon mutants across the PDIM locus, including synthetic function mutants (*ppsC*, *ppsD*, *mas*), transport mutants (*drrA*, *drrB*, *drrC*, *mmpL7*), and acyl activating mutants (*fadD28*) failed to induce expression of type I IFN-responsive gene *ifit2*. P-value by two-tailed t-test for comparisons of Rv with Tn mutants in *eccCb1*, *eccCa1*, *ppsC*, *ppsD*, *drrA*, *drrB*, *drrC*, *papA5*, *mas*, *fadD28*, and *fadD22* were < 0.05, for the comparison of Rv and *mmpL7* was *0*.*05*, and for the comparison of Rv with *lppX*, *pks15*, *Rv2949c*, and *mutT4* were non-significant. (C) A *drrC* transposon mutant was complemented with tetracycline-inducible, chromosomally-integrated *drrC*; *drrC* expression was induced with 50ng/ml or 100ng/ml aTc. While in the absence of inducer *ifit2* expression was substantially decreased relative to wild-type-infected macrophages, induction of *drrC* expression with aTc restored *ifit2* expression. Two-tailed t-test p-value for the comparison of Rv with *Tn*::*drrC* and *Tn*::*drrC*::*ptet-drrC* 0 ng/ml aTc was < 0.01. (D) Infection with wild-type H37Rv, *ppsD* point mutant *ppsD*(G44C) that fails to produce PDIM, or complemented mutants *ppsD*(G44C)rev and *ppsD*(G44C)::*pMVppsD*. While loss of functional *ppsD* in the mutant resulted in loss of *ifit2* expression, restoring *ppsD* function restored *ifit2* production. Two-tailed t-test p-value for the comparison of Rv with *ppsD*mut was < 0.05. (B-D) Mean +/- SD of biological duplicates. (E) Microscopy of macrophages infected with PDIM mutant and complemented strains demonstrates that complementation restores wild-type image analysis phenotypes. J774A.1 macrophages were infected with wild-type, PDIM mutant, or complemented strains. After 3 days of infection, cells were washed, fixed, and stained with DAPI to visualize macrophage nuclei and auramine-rhodamine to visualize *Mtb*. Cells were imaged and CellProfiler automated image analysis was used [29] to quantitate bacterial fluorescent intensity, percent macrophages infected, and macrophage cell count. Mean +/- SD of six biological replicates.

Notably, mutants in pathways for two well-known virulence factors, ESX-1 and PDIM, clustered together by this multiparametric analysis combining imaging and induced cytokine phenotypes. The ESX-1 protein secretion system has been proposed to permeabilize the phagosomal membrane, facilitating recognition of *Mtb* by the macrophage cytosolic surveillance program (CSP) and triggering a type I IFN response [7–10]. On balance, accumulating evidence suggests that this response benefits the bacterium. As expected, we found that ESX-1 mutants failed to induce a type I IFN response (Fig 3A). Surprisingly, PDIM mutants similarly failed to induce a type I IFN response (Fig 3A). Previously, ESX-1 secretion was the only *Mtb* function linked to the macrophage type I IFN response. Although the ESX-1 ATPase EccCa1 has been proposed to bind to a variety of enzymes required for *M*. *marinum* lipid biosynthesis, including some of the enzymes required for PDIM biosynthesis [28], a functional link has not previously been proposed between ESX-1 and PDIM. Our results suggest that PDIM functionally interacts with ESX-1, as it also plays a role in the intracellular events that culminate in induction of type I IFNs.

Critical for *Mtb* growth in the host [11,12], PDIM is a large *Mtb* cell-surface lipid. The chromosomal locus responsible for producing and exporting PDIM encodes machinery for acyl chain production, acyl activation, post-production modification, and transport. Specific functions for PDIM have only recently been proposed, including mediating interactions between the bacterial cell and host plasma membrane [13] and masking MYD88-dependent detection of pathogen-associated molecular patterns (PAMPs) on the mycobacterial surface [14]. Our results indicate that PDIM is additionally required for intracellular processes leading to induction of the macrophage type I IFN response.

We hypothesized that either a distinct PDIM locus gene function (such as function of a single transporter) or fully intact, properly localized PDIM could be required to induce a type I IFN response. To systematically identify the PDIM locus functions required, we profiled expression of type I IFN-induced genes following macrophage infection with mutants that contained disruptions in many genes across the entire PDIM locus (Fig 3B). Mutants defective in each of the functions of the PDIM locus including acyl chain production (*ppsD*, *ppsD*, *mas*), acyl chain activation (*fadD28*), and transport (*drrABC*, *mmpL7*) failed to induce a type I IFN response. The only PDIM locus mutant that induced wild-type levels of type I IFN was *lppX*. Although proposed to be required for PDIM transport [30], *lppX* is in a different operon with *pks1/15* genes [31]. Our results suggest that transporters *drrABC* and *mmpL7* have functions distinct from *lppX* in lipid localization and ultimate function. The loss of a type I IFN response following macrophage infection with all other available mutants in the locus suggest that intact and properly localized PDIM is necessary for induction of type I IFNs.

To confirm that loss of the type I IFN response was in fact attributable to individual genes within the PDIM operons and provide additional confirmation of roles for both PDIM biosynthetic and transport genes, we used two mutant complementation strategies. First, we introduced an integrating, inducible allele of transporter *drrC* to complement *drrC* disruption in the corresponding transposon mutant. Loss of PDIM export was confirmed to be commensurate with what has previously been described for a comparable *drrC* transposon mutant [32] (S5 Fig). Second, we used a published mutant with a single loss-of-function point mutation in biosynthetic gene *ppsD* (*ppsD*(G44C)) and complemented mutant strains with either a chromosomal reversion of the mutation or episomal expression of the wild-type allele [33]; the published loss and restoration of PDIM in the mutant and revertant were confirmed using LC-MS (S6A Fig). Infection with either *Tn*::*drrC* or *ppsDmut* failed to induce the type I IFN response; in both cases, complementation restored the response (Fig 3C and 3D). As expected, the imaging phenotypes of the mutants in infected macrophages also reverted to wild-type by complementation, confirming that PDIM production and transport genes are required for full virulence (Fig 3E). Bulk secretion of IFN-β was more modest than the induced transcriptional response but followed a similar trend for each mutant (S7 Fig).

### PDIM contributes to the type I IFN response by facilitating phagosomal permeabilization

**Fig 4:**
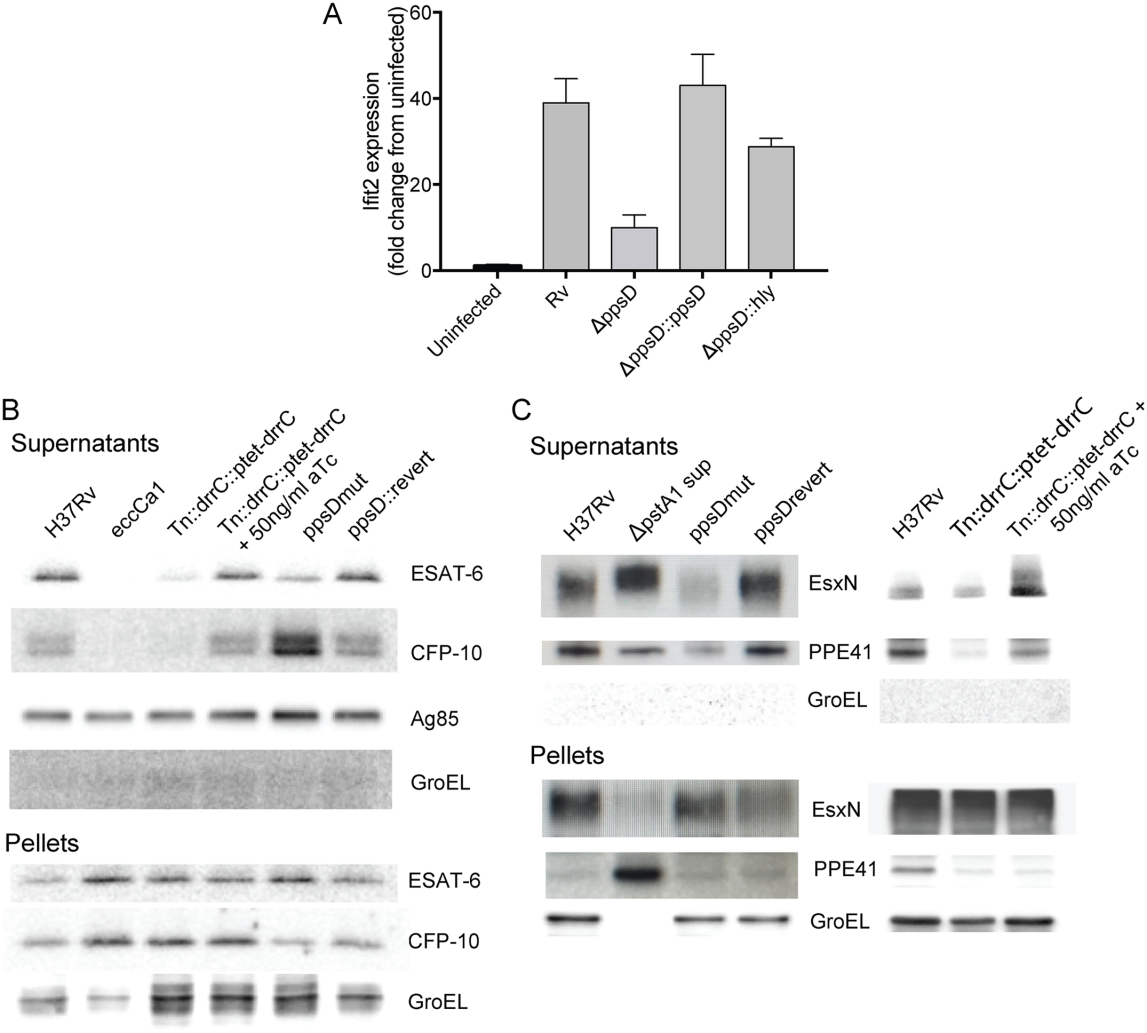
PDIM facilitates phagosome permeabilization and is required for coordinated ESX-1-mediated secretion. (A) Bone marrow-derived macrophages were infected with wild-type *Mtb*, a *ppsD* clean deletion strain (*ΔppsD*), *ΔppsD* complemented with *ppsD*, or the *ΔppsD* expressing listeriolysin O (*hly*) at an MOI of 2:1. RNA was harvested at 24 hours for gene expression analysis by qRT-PCR. Hly-mediated mediated phagosomal permeabilization restores the type I IFN response in the PDIM mutant. Mean +/- SD of biological duplicates. Two-tailed t-test p-value for the comparison of Rv with *ΔppsD* was < 0.001 and for the comparison of Rv with *ΔppsD*::*hly* was non-significant. (B) Western blot analysis of ESAT-6 and CFP-10 secretion in PDIM mutants. Supernatants from wild-type, ESX-1 mutant (*Tn*::*eccCa1*), or PDIM mutants and complements were harvested, concentrated, and probed for ESAT-6, CFP-10, antigen 85 (Sec-secreted control), and GroEL (lysis control). Corresponding pellets were simultaneously lysed and probed for ESAT- or CFP-10 (production control) and GroEL (loading control). Disruption of *drrC* results in loss of ESAT-6 and CFP-10 secretion; inducibly restoring *drrC* expression with 50ng/ml aTc restores ESAT-6 and CFP-10 secretion. Loss of *ppsD* function due to a point mutation in the active site (*ppsD*(G44C)) similarly results in loss of ESAT-6 secretion, but results in enhanced CFP-10 secretion. Reversion of the mutation back to wild-type *ppsD*(G44C)rev restores the wild-type phenotype. (C) Western blot analysis of ESX-5 secretion of EsxN and PPE41 in the *ppsD* point mutant and *drrC* transposon mutant. Supernatants from phosphate-starved wild-type or PDIM mutant and complement were harvested, concentrated, and probed for ESX-5 substrates EsxN or PPE41 and GroEL (lysis control). Supernatant from an ESX-5 hypersecreter strain (*ΔpstA1*) was used for definitive band identification [40]. Loss of *ppsD* function due to the G44C active site point mutation or loss of *drrC* function in the transposon mutant leads to significant loss of secretion of ESX-5 substrates PPE41 and EsxN. In both cases, secretion of EsxN and PPE41 is restored with complementation. Pellets below demonstrate that the *ppsD* mutant and *drrC* transposon mutant do not have a defect in EsxN or PPE41 production.

We next investigated how PDIM might facilitate induction of the type I IFN response. To explore whether PDIM is a PAMP recognized by the macrophage CSP after ESX-1 permeabilizes the phagosomal membrane, we treated macrophages with a liposomal formulation of PDIM. No type I IFN response was elicited even at high concentrations of PDIM (S8 Fig), consistent with recent work suggesting that bacterial genomic DNA is instead the relevant CSP-detected PAMP triggering type I IFNs [8–10]. One step previously described as critical for induction of the macrophage type I IFN response to *Mtb* infection is disruption of the phagosomal membrane, which allows mixing of cytosolic and phagosomal contents and detection of bacterial products by the CSP [7]. To test whether PDIM facilitates phagosomal permeabilization, we sought to determine whether permeabilizing the phagosome by an alternative mechanism would render PDIM dispensable for induction of the type I IFN response. Using a previously described method for creating pores in the phagosomal membrane [7,34], we used listeriolysin O (*hly*) to permeabilize the phagosome in an ESX-1-independent manner. A *ppsD* clean deletion mutant (S6B Fig) was transformed with an integrating plasmid expressing *hly* (S9 Fig). Expression of listeriolysin O in PDIM mutant bacteria indeed restored the type I IFN response (Fig 4A), suggesting that PDIMs contribution to this response is at the level of phagosomal permeabilization. Two other groups have recently reported similar observations using flow cytometry to quantitate phagosomal permeabilization [35,36].

### PDIM is required for coordinated secretion of ESX-1 and ESX-5 type VII secretion system substrates

ESX-1 protein secretion is the only other *Mtb* factor described to be required for phagosomal permeabilization. To determine whether PDIM is required for ESX-1 function itself, we tested whether mutants in PDIM biosynthesis and export were able to secrete the ESX-1 effector ESAT-6, which until very recently had been implicated as the likely pore-forming secreted effector of the ESX-1 system. Using Western blot analysis of bacterial supernatants and pellets, we found that ESAT-6 secretion was indeed impaired in both the *ppsDmut* biosynthesis mutant and the *Tn*::*drrC* export mutant (Fig 4B). ESAT-6 secretion was similarly impaired in strains with clean deletions of *ppsD* and the PDIM biosynthetic enzyme *mas* (S6B and S10 Figs). We then tested whether secretion of the heterodimeric partner of ESAT-6, CFP-10, was similarly impaired in PDIM mutants. Distinct from the phenotype observed for ESAT-6 where its secretion was impaired in all PDIM mutants, CFP-10 secretion was impaired in the *drrC* mutant and *mas* clean deletion, but was enhanced in the *ppsD* mutants (Fig 4B; S10 Fig). Wild-type phenotypes were restored by complementation of the mutations in all cases. Of note, simultaneous analysis of the lysed bacterial pellets indicated that total ESAT-6 and CFP-10 protein production was not affected by the absence of PDIM and, secretion of the Sec substrate antigen 85 was not impacted by the absence of PDIM, suggesting that PDIM does not globally impact secretion.

To determine whether PDIM’s disruption of ESX-1-mediated secretion was generalizable to other ESX secretion systems implicated in virulence [37,38], we next tested whether loss of PDIM disrupts secretion of ESX-3 and ESX-5 substrates. ESX-3 secretion was elicited by iron starvation [39]; ESX-5 was elicited by phosphate starvation [40]. While PDIM mutants did not show impaired secretion of ESX-3 substrates EsxG and EsxH (S11 Fig), the *ppsD* point mutant and *drrC* transposon mutant did demonstrate impaired secretion of ESX-5 substrates PPE41 and EsxN (Fig 4C). Simultaneous detection from bacterial pellet lysates indicated that production of ESX-5 substrates was not changed in either mutant. Thus, while the dependence of secretion on PDIM is not restricted solely to ESX-1 substrates but also impacts the secretion of ESX-5 substrates, it does demonstrate some specificity as it is not generalizable to all ESX secretion systems.

### Two genes that cluster with the ESX-1 and PDIM are required for PDIM production

**Fig 5:**
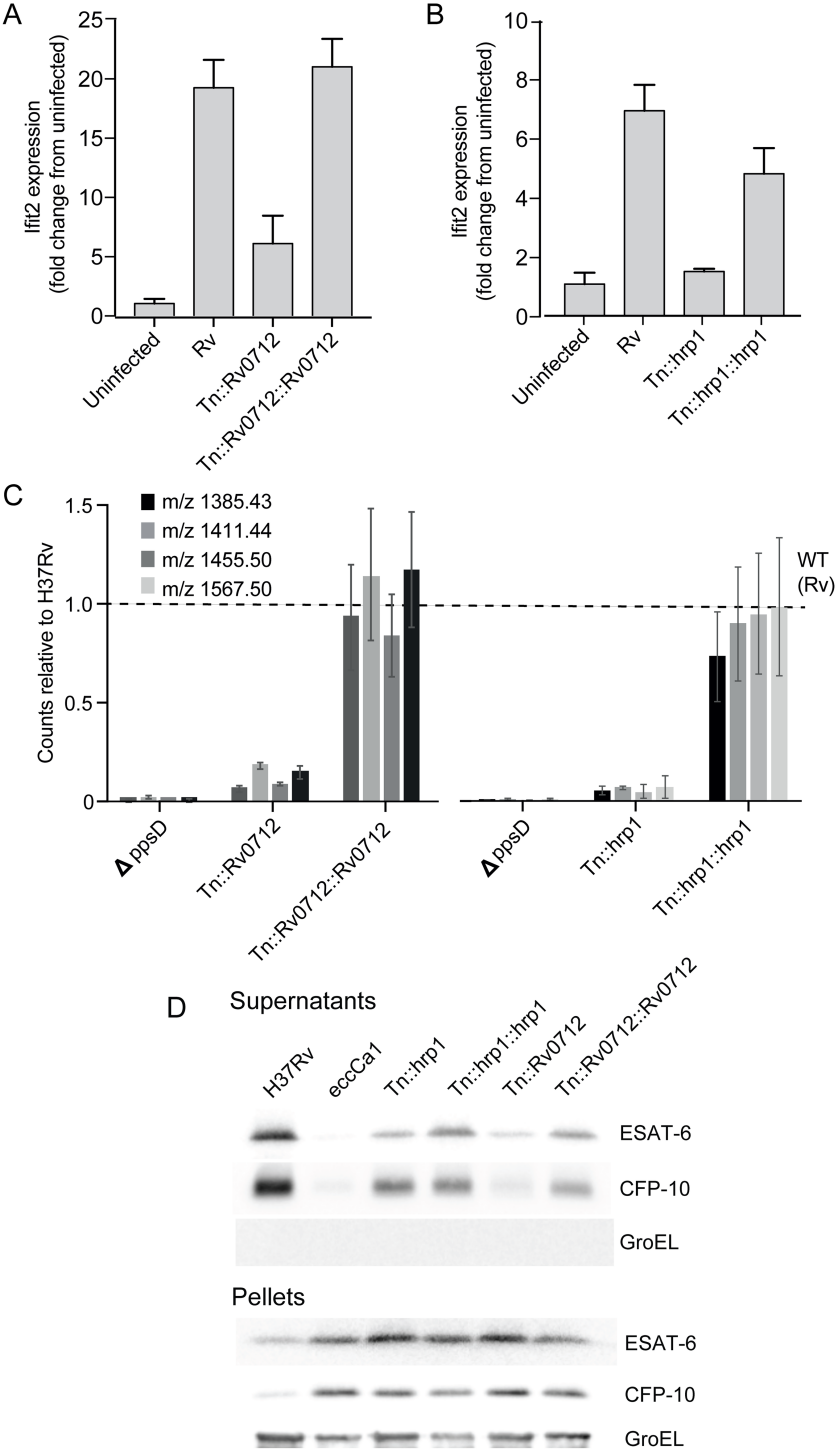
Two additional genes that cluster with PDIM and ESX-1 are required for PDIM production and downstream induction of the type I IFN response. (A-B) The type I IFN response to infection was measured by quantitation of expression of the type I IFN response-representative gene IFIT2. Bone-marrow derived macrophages were infected with wild-type *Mtb*, the *Rv0712* transposon mutant and complemented mutant (A) or *hrp1* transposon mutant and complemented mutant (B). RNA was harvested at 24 hours for gene expression analysis by qRT-PCR. IFIT2 was quantitated relative to control expression of GAPDH. Loss of type I IFN response to infection seen with disruption of *Tn0712* or *hrp1* in each mutant is restored with expression of the respective gene in the complemented strains. (A-B) Mean +/- SD of biological duplicates. Two-tailed t-test p-value for the comparison of Rv with *Tn*::*hrp1* and *Tn*::*Rv0712* was < 0.001. (C) PDIM was quantitated using LC-MS analysis of total cell wall lipid extracts. The indicated strains were grown in tween-free medium; total cell wall lipids were extracted from the bacterial cells. Loss of PDIM production seen in *Rv0712* and *hrp1* transposon mutants was restored with expression of the respective genes in the complemented strains. Mean +/- SD of biological triplicates. Fragmentation patterns for each quantitated species is listed in S1 Text. (D) Western blot analysis of ESAT-6 and CFP-10 secretion in *Rv0712* and *hrp1* mutants. Supernatants from each indicated strain including H37Rv wild-type control and *Tn*::*ecca1* (ESX-1 mutant positive control) were harvested, concentrated, and probed for ESAT-6 or CFP-10, and GroEL (lysis control). Corresponding pellets were simultaneously lysed and probed for ESAT- or CFP-10 (production control) and GroEL (loading control). Disruption of *Rv0712* resulted in diminished secretion of both ESAT-6 and CFP10; loss of secretion was reversed with complementation. Disruption of *hrp1* resulted in diminished ESAT-6 secretion but no change in CFP-10 secretion. Diminished ESAT-6 secretion in the *hrp1* mutant was restored with complementation.

To further test whether mutant clustering by multiparametric analysis can predict novel functional relationships between genes, we next investigated the relationship between the functions of other genes represented in Cluster V and the functions of PDIM and ESX-1. We first sought to confirm that the observed, shared phenotype could be truly accounted for by the transposon-disrupted gene in each mutant by complementing several of the additional 8 mutants that appear in Cluster V with episomal copies of the respective wild-type genes, and tested for reversion to wild-type production of the type I IFN response. Single-gene complementation of two Cluster V mutants, *Tn*::*Rv0712* and *Tn*::*hrp1* (hypoxic response protein 1, *Rv2626c*), restored the type I IFN response (Fig 5A and 5B), suggesting that each of these genes is required for induction of type I IFNs. *Rv0712* is a formylglycine-generating enzyme (FGE), required for activation of type I sulfatase enzymes [41]; however, the physiologic substrates and role of sulfatases in infection is unknown [42]. *Rv0712* has been predicted to be essential in mice based on Tn-Seq experiments [43]. *hrp1* is part of the DosR regulon, which is highly expressed upon exposure to nitric oxide or hypoxic conditions, and proposed to play a role in dormancy [44–46]. Hrp1 is a secreted protein with two cystathionine beta synthase (CBS) domains [47] but its biological function and role in infection is also unknown. Neither gene has previously been linked to mycobacterial lipids, ESX-mediated secretion, or the macrophage type I IFN response.

Based on its phenotypic clustering, we hypothesized that *Rv0712* and *hrp1* could be required for PDIM biosynthesis thereby facilitating ESX-1 secretion and phagosomal permeabilization, for ESX-1 secretion independent of PDIM, or for an altogether independent function influencing the type I IFN response, such as the production or exposure of a bacterial PAMP or a role in host CSP signaling. Using quantitative mass spectrometric analysis of total cell wall mycobacterial lipids [48], we determined that in fact, both *Tn*::*Rv0712* and *Tn*::*hrp1* produced significantly less PDIM than wild-type H37Rv (Fig 5C; S12A and S12B Fig). PDIM production in the mutants was restored with complementation. We then tested whether the observed loss of PDIM in the mutants would correlate with changes in secretion of ESX-1 substrates. Secretion of ESAT-6 was diminished in both the *hrp1* and *Rv0712* mutants and restored in the complemented strains (Fig 5D); secretion of CFP-10 was diminished in the *Rv0712* mutant but unchanged in the *hrp1* mutant. Thus this multiparametric clustering successfully predicted that these two genes would functionally interact with PDIM and ESX-1 in their induction of the macrophage type I IFN response.

## Discussion

A deeper, systematic understanding of the *Mtb* host-pathogen interface is a necessary prerequisite to developing a new generation of anti-TB therapeutics that target the functions most critical for bacterial survival and growth in the host setting. We used a multiparametric analysis combining imaging phenotypes and macrophage cytokine responses to infection to systematically identify *Mtb* mutants impaired for intracellular survival and predict novel functional relationships among subsets of those genes based on shared complex phenotypes. We have demonstrated the strength of this approach by demonstrating its ability to identify a previously unknown relationship between two well-known virulence factors, ESX-1 and PDIM, and to assign function to two genes not previously described to be important for intracellular survival, now linking them to the function of these two known virulence factors. We anticipate that our approach can be easily applied to identify additional functional relationships between genes. Given the increasing ability to perform systematic high-throughput profiling of cellular phenotypes such as infection using arrayed libraries of bacterial mutants, we propose that this type of multi-parametric analysis can be extended to other pathogens and additional phenotypes to identify genes that are functionally linked and assign functions to genes of unknown function. Given the high percentage of bacterial genes with unknown function in many pathogens including *Mtb*, the ability to functionally link known and unknown genes is a powerful approach for building our understanding of pathogenesis.

During *Mtb* infection, the mechanisms that the bacterium requires to ensure its own survival are critical to determining the outcome of infection. Disruption of the phagosomal membrane, which permits mixing of phagosomal and cytosolic contents, has complex downstream consequences, simultaneously triggering pro-inflammatory and anti-inflammatory programs in the macrophage [9]. Proposed benefits of this permeabilization for the bacterium include increased access to macronutrients and micronutrients. Additionally, this permeabilization leads to induction of the type I IFN response upon CSP detection of *Mtb* gDNA. Although the direct pathogenic relevance of type I IFNs in *Mtb* infection remains a matter of active scientific debate, growing evidence suggests that on balance, type I IFNs benefit *Mtb* in the bacterial/host standoff [49,50].

The role of the type I IFN response and in particular the molecular details of the host factors that lead to induction of type I IFNs and downstream effects have been an active area of recent investigation [7–10,51]. While details of the events on the host side of the *Mtb* and macrophage interaction that culminate in production of type I IFNs have been well-elucidated, until very recently, ESX-1-mediated phagosomal permeabilization was the only mycobacterial event implicated [7]. Two recent reports have suggested that PDIM is involved in phagosomal permeabilization [35,36]. Here, we also present evidence that is consistent with the finding that the *Mtb* virulence lipid PDIM is a second *Mtb* factor contributing to the permeabilization of the phagosome. Our data additionally suggest that this effect is at least in part due to its impact on ESX-mediated secretion. Intact ESX-1 secretion has been shown to be required for phagosome permeabilization [7,52,53] with ESAT-6 thought to be the responsible effector [15,16]. Whether ESAT-6 is truly the secreted ESX-1 effector that mediates phagosomal disruption has very recently been called into question [54]. In that report, at non-acidic pH, purified ESAT-6 was unable to lyse membranes without residual detergent from the preparation. In contrast, at acidic pH, ESAT-6 alone was sufficient to lyse membranes, consistent with earlier reports. Whether PDIM serves a detergent function to facilitate ESAT-6-mediated phagosomal lysis at relatively non-acidic pH in the early phagosome as suggested by Augenstreich et al., whether focal acidic conditions at points where the *Mtb* membrane meets the phagosomal membrane facilitate ESAT-6-mediated membrane lysis, or whether an entirely distinct secreted ESX-1 effector such as EspC is responsible for phagosomal membrane disruption as suggested in the discussion by Conrad et al., remains to be determined.

Although PDIM and ESX-1 are two of the best-described *Mtb* virulence factors, until very recently they had not been understood to contribute directly to the same pathogenic process. A potential role for ESX-1 in regulating the essential cell wall mycolic acids has been suggested [28], and genetic disruptions of the ESX-1 and ESX-5 systems have been described to render the mycobacterial cell wall more susceptible to detergent and antibiotic-mediated disruption [55,56]. In contrast, the reverse interaction, i.e., the impact of PDIM on secretion, has been less clear. Though loss of PDIM has been reported to abrogate the essentiality of individual ESX-5 secretion system components during macrophage infection [10], the concept that a surface lipid facilitates protein secretion has not previously been proposed for *Mtb* or any other bacteria.

The complete mechanistic basis of type VII secretion is an ongoing area of active investigation, and the current model for how ESX-mediated secretion occurs is largely limited to bacterial cytosolic and inner membrane events [52]. In the context of what is understood about ESX-mediated secretion, our determination that PDIM is required for secretion of some ESX substrates *i*.*e*., ESAT-6, PPE41 and EsxN but not others *i*.*e*., CFP-10, EsxG, and EsxH, is intriguing. The position of PDIM in the cell envelope raises the question of whether PDIM facilitates the steps for secretion of individual substrates beyond the inner membrane, such as transport across outer layers of the cell, dissociation of the heterodimeric secretion pairs, or release from the cell surface. This conjecture would be consistent with the observed distinct secretion phenotypes for different substrates. The disparate CFP-10 secretion phenotypes observed for different PDIM mutants suggest that distinct parts of the lipid molecule may interact with individual substrates. A role facilitating transit across the outer layers of the cell or release from the cell surface would also explain why mycobacteria, including *Mycobacterium smegmatis*, that do not produce PDIM have no secretion defect. While the core ESX-1 secretion machinery is nearly identical between *Mtb* and *Msmeg*, the outer envelope has significantly less similarity. Given that the interdependence of ESX-1 substrates for secretion has made studying the role of individual effectors in both secretion and pathogenesis challenging, strains with distinct secretion phenotypes offer an opportunity for additional dissection of the role of individual effectors in pathogenesis.

Although the uniquely rich repertoire of *Mtb* lipids has long been recognized as important for pathogenesis, ascribing defined molecular roles to those lipids has proven challenging, in part because of the more limited range of tools available for studying lipids relative to proteins or nucleic acids. Here, we ascribe roles to PDIM as facilitating both phagosomal permeabilization and secretion of individual ESX substrates. More broadly, our results illustrate a novel relationship in which bacterial protein secretion is dependent upon a bacterial lipid at the cell surface. As new technologies including comparative lipidomics [48] open the door to understanding the molecular functions of individual lipids, we anticipate that paradigms for the role of bacterial lipids in infection will fundamentally change.

Our multiparametric clustering additionally facilitated the determination that two additional genes required for virulence, *Rv0712* and *hrp1* act upstream of PDIM production in facilitating the type I IFN response. While additional work remains to fully determine how *Rv0712* and *hrp1* contribute to complex lipid production, the identification of a relationship between each of these genes, PDIM production, and ESX-mediated secretion confirms that clustering by host and pathogen phenotypes can suggest functions for genes with an unknown role in virulence. Specifically considering *Rv0712*, our work suggests that type I sulfatase activity is important for *Mtb* infection of macrophages. Notably, neither our screen nor previous screens have identified individual sulfatases as essential in host cells, suggesting possible redundancy among the type I sulfatases with *Rv0712* potentially acting as a functional regulator. While PDIM and sulfated lipids share the common methylmalonyl CoA precursor, the relationship between these two lipids has previously been shown to be inversely coupled through anabolic pathways [57]. The requirement of *Rv0712* for PDIM production suggests a more complicated relationship of PDIM and sulfate metabolism pathways.

We suggest that the dataset arising from this multiparametric cluster analysis offers the opportunity for the discovery of other novel functional interactions among genes required for infection. For example, as the macrophage type I IFN response was a major contributing factor in the generation of Cluster V, further investigations of mutants in this cluster may reveal bacterial factors that interact more directly with the host CSP that triggers the type I IFN response. Similarly, as production of cytokines such as TNF-α, MMP9, and MIP-1α was a major factor in formation of Clusters I-IV, genes involved in these clusters are likely mostly involved in host interactions downstream from cell-surface and phagosomal PRRs, including TLRs. Yet the patterns of induction or suppression of these cytokines are distinct in the different clusters of mutants. Thus, this analysis presents the opportunity to dissect the different sets of bacterial factors that interact with and modulate the responses of these PRRs in distinct and complex ways. Particularly given that the bulk of work studying PAMP/PRR interactions has been undertaken with purified PAMPs, we thus further anticipate that the generated dataset characterizing the comprehensive infection of macrophages with *Mtb* mutants will offer opportunities for unraveling the complexities of PAMP/PRR interactions within the context of an intact bacterium, in which each interaction contributes to the ultimate response.

Finally, these results and approach have implications for future drug development. With increasing interest in not simply targeting essential factors of *Mtb* but also alternative strategies that take into account host responses, understanding the impact of targeting different bacterial factors on local innate immune responses will be important.

## Materials and methods

### Bacterial strains and growth conditions

The transposon mutant library was made in *Mtb* strain H37Rv. For genetic manipulations, *Mtb* was growth in Middlebrook 7H9 broth (Difco) supplemented with Middlebrook OADC (BD), glycerol 0.2%, and tween-80 0.05%. *Mtb* strains *ppsD*(G44C), *ppsD*(G44C)revert and *ppsD*(G44C)pMV::*ppsD* were previously published strains [33]. The inducible *drrC* construct was generated by cloning the *drrC* gene into pMC1S [58]. The hly-expressing plasmid was generated by cloning *Listeria hly* into pMC1S. The *ppsD* and *mas* clean deletion strains were generated using mycobacterial recombineering to replace the entire *ppsD* or *mas* gene with a hygromycin cassette [59]. *Tn*::*Rv0712* and *Tn*::*hrp1* were selected from the arrayed, annotated transposon library and complemented with each individual gene in the episomal, constitutively expressing vector JP118 [39]. Gene-specific primers are listed in S1 Text.

### Construction of an arrayed, annotated transposon mutant library

*Mtb* strain H37Rv was transposon mutagenized using a Himar-based transposon according to the protocol described in Sassetti *et al* [60]. Following selection on kanamycin-containing plates, approximately 26,000 individual colonies were selected and arrayed as glycerol stocks in 96-well plates. Colony PCR was then performed, using a two-step process with one primer internal to the transposon and one random primer for each PCR step to identify each insertion site. Each PCR product was then subjected to Sanger sequencing. Arrayed library mutants were selected after processing the data with a BLAST alignment-based script that incorporates a number of criteria, including Sanger read sequence quality, the uniqueness of genome sequence match, location of alignment within the sequence read and e-value of the BLAST match. In cases of genes demonstrating multiple insertion sites, priority was assigned to insertion sites greater than 10 nucleotides into an ORF or more than 50 nucleotides upstream of the stop. Ultimately 2660 transposon mutants, each with insertion in a different gene, were selected and arrayed in 28 96-well plates.

### Characterization of library growth in vitro

The Tn mutant library was inoculated into 96-well plates containing 100μL Middlebrook 7H9 broth supplemented with Middlebrook OADC, glycerol 0.2%, and tween-80 0.05%. Plates were then incubated at 37°C. Every 2–3 days, wells were resuspended by pipetting and OD600 was determined using a SpectraMax plate-reader (Molecular Devices). Growth rates (υ) were calculated for each mutant by fitting a sigmoid function to the growth curve (OD600 over time) and estimating the slope. Most mutants in the library grew similarly in 96-well plates, and attenuated mutants did not grow differently than all mutants (S13A and S13B Fig).

### High-content assay protocol

Full assay development details are in S1 Text. Briefly, transposon mutants were grown in 96-well plates in 7H9 media with OADC, glycerol, and tween-80 supplementation. On the day of infection, plates were centrifuged to pellet bacteria and media was removed. Pellets were resuspended in an equal volume of PBS, and plates were again centrifuged to pellet bacteria. Supernatants were removed, and cells were resuspended thoroughly in PBS. A low-speed spin was performed to pellet cell clumps. The supernatant was moved to another 96-well plate, and OD600 for each well was recorded. The average bacterial number per ml across the plate was calculated, and bacteria were added to DMEM with 20% heat-inactivated horse serum (HS) to give an average number of CFU/ml of 62,500 (averaged across all wells on the plate). The bacteria in DMEM/HS were then added to J774A.1 macrophages in 96-well plates to give an average MOI of 1:1. Control bacteria were prepared in parallel, and added to macrophages at an MOI of 2:1, 1:1, 1:2, and 1:4. After 4 hours for phagocytosis, media was aspirated, infected cells were washed once with PBS, and media was added back. Cells were incubated for 3 days, then media was aspirated off. Cells were again washed with PBS to remove extracellular bacteria, then fixed with 4% paraformaldehyde. Wells were then stained with auramine-rhodamine to visualize bacteria and DAPI (Sigma) to visualize macrophage nuclei. Plates were imaged on an ImageXpress high-content microscope (Molecular Devices). Images were then analyzed using CellProfiler automated image analysis [29].

### CellProfiler automated image analysis pipeline

The basic operation of the image analysis workflow is similar to that used previously [17]. Briefly, a pre-processing CellProfiler pipeline was used to determine the illumination correction of each fluorescent channel. A second pipeline then used the results of the first pipeline to correct each channel for uneven illumination, followed by detection and exclusion of fluorescent debris, and collection of whole-image features from the corrected images including the green fluorescent pixel intensity and area integrated across the field of view, the macrophage number per field of view and the percentage of macrophages that are infected. This pipeline additionally collected a variety of image-based features for each auramine-rhodamine-labeled mycobacterial clump and the associated macrophage DNA-labeled nuclei, in order to produce rich, quantitative profiles in an unbiased manner. These features included mycobacterial and nuclear size and shape, intensity, and texture statistics. All pipelines used for this paper are publically available at http://www.cellprofiler.org/published_pipelines.shtml.

### Statistical analysis of screening data

The features extracted from the image analysis pipeline were used to select hits as well as identify phenotypically distinct groups of mutants within the hits. By analyzing the features in the pilot screen, we identified three features (macrophage count, *Mtb* fluorescence intensity, percentage of infected macrophages) to be best predictive of CFU (S1 Text). To account for the small differences in MOI across replicates, the residues of the feature values regressed against the MOI were used in all further analyses. We combined the three features into a single readout by computing a linear combination for which the variance in the data was maximized (i.e. the first principal component “PC1” of the data). We found that PC1 was a better predictor of CFU compared to the individual features (S1C Fig, S1 Text) or a combination of all measured features (S1D Fig) and therefore used it to select mutants for follow up experiments.

### Cytokine profiling of supernatants from macrophages infected with 361 screen hits

The 361 attenuated *Mtb* Tn mutants were grown in 4 96-well plates to mid-log phase. J774A.1 macrophages were seeded in 96-well plates overnight. Cells were then infected with the attenuated mutants or controls in a 96-well format, prepared as described above. The average MOI across the plate was 1:1. Infection was allowed to progress for 3 days (or 48 or 72 hours for the timecourse), then supernatants were harvested and used in a Luminex assay for detection of 34 multiplexed cytokines according to the manufacturer’s protocol.

### Multiparametric analysis for mutant clustering

All mutants included in the primary screen were ranked in deciles for each of the three imaging features: bacterial fluorescence intensity, macrophage cell count, and percent macrophages infected. Host cytokine were clustered in an unsupervised manner into four groups based on behavior across all hit mutants. The 113 mutants eliciting cytokine responses most distinct from wild-type infection were then clustered using hierarchical clustering with Pearson correlation as the similarity metric. For the clustering, each mutant was represented by a 7-dimensional feature vector representing, both, imaging (3 features) and cytokine response (4 feature groups).

### Western blotting for ESX secretion assays

*Mtb* strains were grown in Middlebrook 7H9 broth (Difco) supplemented with Middlebrook OADC (BD), glycerol 0.2%, and tween-80 0.05% to mid log-phase. For ESX-1 blotting, an equal number of cells from each culture were pelleted by centrifugation and resuspended in Sauton’s media supplemented with OADC and glycerol for 24 hours. For ESX-3 blotting, an equal number of cells from each culture were pelleted by centrifugation, washed three times in chelated Sauton’s medium [61], and resuspended in chelated Sauton’s medium for 48 hours. For ESX-5 blotting, an equal number of cells from each culture were pelleted by centrifugation, washed and resuspended in medium phosphate Sauton’s medium (250μM phosphate) with tween for 48 hours, then washed and resuspended in low phosphate Sauton’s medium (25μM phosphate) without tween for 48 hours (modified from [40]). Supernatants were then harvested and proteins were concentrated 500-fold. Concentrated supernatants were run on an Tris-glycine gel and probed with antibodies to ESAT-6 (Abcam), CFP-10 (Abcam), Antigen 85 (Abcam), GroEL (Abcam), EsxGH [61], a kind gift of Dr. Jennifer Philips, Washington University, EsxN, or PPE41. Blots were stained with Ponceau prior to antibody detection to ensure equal protein loading of all lanes. Pellets were lysed by bead beating in protein extraction buffer (50mM TrisCl, pH 7.5, 5mM EDTA, 1mM 2-mercaptoethanol, protease inhibitor (Amresco)) prior to use for Western blotting.

### Production of EsxN and PPE41 antisera

Full-length *esxN* was amplified from *M*. *tuberculosis* Erdman genomic DNA by PCR with primers esxNHisF2 (5’gcattcatgacgattaattaccagttcgggga3’) and esxNHisR2 (5’ gcatctcgagggcccagctggagccga3’), digested with BspHI and XhoI and cloned in pET28b+ (Novagen) between the NcoI and XhoI restriction enzyme sites to generate pET28-EsxNHis_6_ encoding EsxN with a C-terminal His_6_ tag. A plasmid for co-expression of PPE41-His_6_ and PE25 was described previously [62]. Recombinant proteins were produced in *E*. *coli* BL21(DE3) and purified by Ni^2+^-NTA affinity chromatography (Qiagen). PPE41-His_6_ was bound to the column under native conditions in 20 mM HEPES buffer, 300 mM NaCl, pH 7.8 and eluted in 20 mM HEPES buffer, 500 mM NaCl, pH 7.8 containing 50–150 mM imidazole. EsxN-His_6_ was purified under denaturing conditions; protein was bound to the column in 20 mM sodium phosphate buffer, 500 mM NaCl, 6 M guanidine hydrochloride, pH 7.8 and eluted in 20 mM sodium phosphate buffer, 500 mM NaCl, 8 M urea, pH 4. Contaminant proteins that co-purified with EsxN-His_6_ were removed by passing eluted fractions through a 50 kDa cut-off Amicon Ultra centrifugal filtration unit (Millipore). Polyclonal antisera against purified EsxN-His_6_ and PPE41-His_6_ proteins were generated in rabbits by Pierce Custom Antibodies (Thermo Scientific) using TiterMax Gold adjuvant (Sigma).

### Macrophage infections for RNA isolation and qPCR

Murine bone marrow-derived macrophages (BMDM) were prepared as previously described [17] from C57BL6 mice (Jackson Laboratories). BMDM were seeded in 24 well plates overnight in DMEM with 20% FBS and 25ng/ml rmM-CSF (R and D Systems). *Mtb* strains were grown to mid-log phase, then used to infect BMDM at an MOI of 2:1. After 4 hours of phagocytosis, cells were washed once with PBS and media was added back. RNA was harvested 24 hours after infection with TRIzol (ThermoFisher Scientific) and prepared according to the manufacturer’s protocol. cDNA was prepared using SuperScript III (ThermoFisher Scientific) according to the manufacturer’s protocol. qPCR was performed using primers specific to the indicated genes.

### Total cell wall PDIM quantitation

Liquid chromatography-mass spectrometric quantitation of PDIM was performed on total cell wall extracts of *Mtb* according to published protocols [48]. In brief, *Mtb* strains were grown in 7H9 supplemented with Middlebrook OADC (BD) and glycerol 0.2%. Cells were then pelleted and extracted in chloroform:methanol and an equal quantity of total lipids were run through an established LC-MS protocol using an Agilent Technologies 6520 Accurate-Mass Q-Tof and a 1200 series HPLC system with a Monochrom diol column [48]. PDIM species were identified using positive mode MS based on predicted retention time [48], highly accurate mass matching to known PDIM species as listed in the figures, and confirmed based on collision-induced dissociation mass spectrometry with major fragments as listed in S1 Text.

### Ethics statement

Protocols for care and use of mice for bone marrow macrophage preparation were approved by the Massachusetts General Hospital Institutional Animal Care and Use Committee. The approved protocol number is 2007N000048. The procedure for euthanasia approved as part of this protocol is in accordance with American Veterinary Medical Association Guidelines for the Euthanasia of Animals.

## Supporting information

S1 Text Supplementary methods. Supplementary Methods includes detailed imaging assay development methods, image analysis details for the training set, pilot screen, gene-specific primers, and collision-induced dissociation fragment information.
(DOCX)

S1 Fig Determination of optimal imaging output metrics and assay performance in a pilot screen. (A) *Mtb* strain H37Rv was used to infect J774A.1 cells simultaneously for imaging and for colony-forming units (CFU) determination. Cells were treated with isoniazid (1μg/ml) or rifampicin (0.025μM or 0.25μM). (B) Comparison of individual imaging outputs and CFU for six growth-impaired mutants identified in the pilot screen. Five out of six were confirmed attenuated by CFU (data shown for the five attenuated mutants) (C) Transposon mutants were ranked (x-axis) by one of four metrics: percent macrophages infected, *Mtb* fluorescence intensity *(Mtb* FI), macrophage cell count, or a primary component analysis (PCA) incorporating all three metrics, against percentage of mutants confirmed to have impaired intracellular growth by CFU (y-axis). PCA best distinguished true positives. (D) Transposon mutants were ranked (x-axis) by their score in the three-feature PCA (“PCA 3 features”) or a PCA incorporating data for all 616 imaging features against the percentage of mutants confirmed by CFU (y-axis). The three-feature PCA better distinguished true positives. (E) Replicate reproducibility of the three individual imaging metrics for the full screen.
(PDF)

S2 Fig Flow chart of mutant screening and analysis. (PDF)

S3 Fig Cytokines used to analyze the macrophage response to infection with hit mutants. To determine whether the dynamic range for each measured cytokine was broad enough to allow meaningful interpretation of mutant results falling between wild-type infected cells and uninfected cells, we compared the normalized cytokine values for wild-type-infected and uninfected cells. Shown are normalized Z-scores of detection of the indicated cytokines of uninfected (red) and control-infected (blue) cells. Values represent +/- SD for three independent replicates.
(PDF)

S4 Fig The macrophage response to infection differs significantly across hit mutants. (A) Macrophages were infected with the 361 individual mutants. Supernatants were collected after 3 days of infection and cytokines were measured by Luminex multiplexed cytokine quantitation. The heatmap (red-blue) shows the 113 mutants inducing at least a two-fold difference in two cytokines from H37Rv wild-type-infected macrophages. (B) The input OD600 and image analysis PC1 (purple-green) for each of the 113 mutants plotted below shows that the cytokine clustering does not correlate with either parameter. (C) Hierarchical clustering of the 12 cytokines into 4 groups based on Pearson correlation for behavior across all hit mutants. (D) correlation distance for inter-cluster and intra-cluster comparisons. In all cases, intra-cluster comparisons have a smaller correlation distance than inter-cluster comparisons.
(PDF)

S5 Fig Raw mass spectra for *drrC* mutant and complement. Bacteria were grown to late log phase in 7H9 with OADC, glycerol, and tween 0.05%. Cells were then pelleted, and supernatant was harvested, sterile filtered, and extracted with hexanes. Hexane extracts were then extracted with water 5 times to reduce background tween contamination. LC-MS was then performed on the extracts according to established protocols [48]. Raw mass spectra were extracted over the retention time where PDIM species (as confirmed by collision-induced dissociation fragmentation patterns) ran.
(PDF)

S6 Fig Raw mass spectra for ppsD and mas mutants. Bacteria were grown to OD600 0.6 +/- 0.1 in media lacking tween. Cells were then pelleted, and total cell-wall lipids were extracted in chloroform:methanol. LC-MS was performed on the extracts according to established protocols [48]. Raw mass spectra were extracted over the retention time where PDIM species (as confirmed by collision-induced dissociation fragmentation patterns) ran. A. Spectra for the H37Rv and the ppsD point mutant strain (*ppsD*(G44C)) and chromosomal reversion. B. Spectra for H37Rv and the *ppsD* and *mas* clean deletions and complements.
(PDF)

S7 Fig IFN-β secretion into culture supernatants of infected macrophages. Bone marrow-derived murine macrophages were infected with the indicated strains at an MOI of 2:1. After a 4 hour phagocytosis step, cells were washed to remove remaining extracellular bacteria and media was added back. Supernatants were harvested for ELISAs at the indicated times after the initiation of infection. Mean +/- SD for 3 replicates.
(PDF)

S8 Fig Macrophage treatment with PDIM does not induce a type I IFN response. Bone marrow-derived macrophages were stimulated with either LPS (100ng/ml) or with the indicated doses of purified PDIM. Cells were harvested at the indicated time points for RNA extraction and cDNA preparation. Real-time qPCR was then used to quantitate expression of type I IFN-responsive gene CCL5. Gapdh was used as a normalizing control for each sample.
(PDF)

S9 Fig Hly is detectable from *ΔppsD*::*hly* in the context of infected macrophages. Macrophages were infected with *ΔppsD* or *ΔppsD*::*hly* at an MOI of 2:1. Mixed macrophage and bacterial RNA was harvested 24 hours after infection for cDNA preparation. PCR with primers specific to *hly* was then performed. *Hly* transcript was detectable from cDNA prepared with reverse transcriptase (+RT) but not from the control sample without reverse transcriptase added (-RT).
(PDF)

S10 Fig Western blot analysis of ESAT-6 and CFP-10 secretion in *ppsD* and *mas* clean deletion mutants. Supernatants from wild-type, ESX-1 mutant (*eccCa1*), or PDIM mutants and complements were harvested, concentrated, and probed for ESAT-6 or CFP-10, antigen 85 (Sec-secreted control), and GroEL (lysis control). Corresponding pellets were simultaneously lysed and probed for ESAT- or CFP-10 (production control) and GroEL. Disruption of *mas* results in loss of ESAT-6 and CFP-10 secretion; restoring mas on a constitutively expressing episomal plasmid restores the wild-type phenotype. The *ppsD* clean deletion mutant shares the phenotype of the *ppsD* point mutant (*ppsD*(G44C)) shown in Fig 4; ESAT-6 secretion is diminished and CFP-10 secretion is enhanced. Complementation with an episomal plasmid constitutively expressing the operon including *ppsD* restores the wild-type phenotype.
(PDF)

S11 Fig PDIM is not required for EsxG and EsxH secretion. Bacteria were grown to mid-log phase, then bacteria were washed twice in Sauton’s medium that had been pre-chelated. After 48 hours of growth, cells were pelleted and supernatants were harvested, concentrated, and run on a Tris-glycine gel. Blots were probed with antibody to EsxGH (gift of Dr. Jennifer Philips, Washington University), and GroEL (lysis control). Corresponding pellets were simultaneously probed for EsxGH and GroEL.
(PDF)

S12 Fig Raw mass spectra for *hrp1* and *Rv0712* mutants. Bacteria were grown to OD600 0.6 +/- 0.1 in media lacking tween. Cells were then pelleted, and total cell-wall lipids were extracted in chloroform:methanol. LC-MS was performed on the extracts according to established protocols [48]. Raw mass spectra were extracted over the retention time where PDIM species (as confirmed by collision-induced dissociation fragmentation patterns) ran. A. Raw spectra for H37Rv, the *ppsD* clean deletion (PDIM negative control), *Tn*::*Rv0712* and *Tn*::*Rv0712* complemented with an episomal plasmid constitutively expressing the gene. B. Spectra for H37Rv, the *ppsD* clean deletion (PDIM negative control), *Tn*::*hrp1*, and *Tn*::*hrp1* complemented with an episomal plasmid constitutively expressing the gene.
(PDF)

S13 Fig *Mtb* transposon mutant library growth in liquid culture and high-content imaging assay development. The *Mtb* transposon mutant library was grown in liquid culture for 14 days. Growth was assessed every 2–3 days by OD600 after mixing. A ν value for growth in liquid culture was then calculated for each mutant. (A) Biological replicate correlation. (B) Histogram of ν values for screen hits or non-hits. The calculated value for each mutant represents the mean of two independent replicates. Screen hits and non-hits have similar growth rates in liquid culture. (C-D) Wild-type *Mtb* strains H37Rv or H37Rv expressing RFP were grown to the shown densities, washed in PBS, and used to infect J774A.1. After a 4 hour phagocytosis, cells were washed to remove any unengulfed *Mtb*. (C) H37Rv::RFP infected cells were stained with auramine-rhodamine (A-R). Red and green fluorescence were compared to determine how well A-R staining identified *Mtb*. (D) Cells infected at the given MOIs were incubated for 3 days. Cells were then washed, fixed, and macrophage nuclei were stained with DAPI. Plates were then imaged and nuclei were quantitated using automated image analysis. Two-fold differences in MOI significantly change macrophage survival. (E) Following infection with H37Rv, macrophages were lysed and plated for CFU. Input OD600 was found to correlate well with quantitation of internalized *Mtb*.
(PDF)

S1 Table Arrayed *Mycobacterium tuberculosis* transposon mutant library with annotation of mutants included in the final high-content screen analysis. (XLSX)

S2 Table High-content imaging screen hits. (XLSX)

S3 Table Selected screen hits retested by CFU in comparison with wild-type *Mtb*. (XLSX)

S4 Table Categories of screen hits in the context of what is known of *Mtb* in the host. (XLSX)

S5 Table Multiparametric clusters of hit mutants. (XLSX)
