# Supplementary material for: Systematic, multiparametric analysis of Mycobacterium tuberculosis intracellular infection offers insight into coordinated virulence
Source: PLoS Pathog. 2017 May 15;13(5):e1006363. doi: 10.1371/journal.ppat.1006363 (PMC5444860; doi:10.1371/journal.ppat.1006363)
Supplement: S1 Text — Supplementary Methods includes detailed imaging assay development methods, image analysis details for the training set, pilot screen, gene-specific primers, and collision-induced dissociation fragment information. (DOCX) [file ppat.1006363.s001.docx]

**S1 Text: Supplementary Methods.**

**Imaging assay development**

In order to adapt our previous high-content assay used for small molecule screening in macrophages [[1](#_ENREF_1)], we overcame three major technical challenges. First, the wild-type *Mtb* strain used in our previous work expressed GFP and was readily visualized, unlike the Tn mutants, which are not fluorescently marked. We developed a high-throughput compatible auramine-rhodamine (A-R) staining protocol to visualize intracellular mycobacteria. After fixing cells with 4% paraformaldehyde (PFA), PFA was removed, and cells were washed with PBS. A 1:10 dilution of auramine-rhodamine stain in water (TN Fluorescent Stain Kit T, BD) was added to cells, which were incubated for 25 minutes. The rest of the staining protocol was followed according to the manufacturer’s recommendations. After the final step, DAPI 1.25μg/mL in PBS was added to the cells. The reliability of the A-R stain was validated using macrophages infected with RFP-expressing *Mtb* (**S13C Fig**).

Second, initial measurements of pixel fluorescence intensity and bacterial area using our prior CellProfiler pipeline [[1](#_ENREF_1)] revealed some non-specific A-R staining of macrophage nuclei and cell bodies despite protocol optimization. Our pipeline was thus adapted to eliminate non-specific detection of macrophage nuclei and cell bodies, thereby allowing more accurate quantitation.

Third, unlike a single uniform culture used for small molecule screening, transposon mutants growing in 96-well plates are inoculated at different densities (depending on their density in the source plate) and are thus at different optical densities (OD_600_) on the day of screening. This heterogeneity is potentially problematic given that the course of intracellular infection and host cell fate differs based on the dose of infecting bacteria relative to host cell numbers (defined as the multiplicity of infection, MOI). Higher MOIs dispose the macrophages to necrotic cell death [[2](#_ENREF_2)] rather than to a stand-off between bacteria and macrophage or to protective apoptosis [[3](#_ENREF_3),[4](#_ENREF_4)]. Given that normalizing the MOI for infections with 2660 individual mutants was not technically feasible, we sought a simpler solution to this heterogeneity. First, we confirmed that OD_600_ of the bacteria was in fact a reliable indicator of intracellular bacterial CFU after uptake and thus could be used to calculate MOIs for mutants in this format (**S13D Fig**). We then determined the range of MOIs that could be tolerated during infection without changing the biology of infection. A four-fold increase in the MOI was determined to alter the outcome of infection, with significantly more macrophage death by day 3 post-infection (**S13E Fig**).

We found that most of the 2660 mutants had similar growth rates in 96-well liquid culture, making them amenable to high-throughput screening (**S13A-B Fig**). Growing all mutants in this format and then converting from OD_600_, we targeted an MOI of ~1 for infection for the majority of mutants. To ensure accurate assessment of each individual mutant phenotype, mutants were binned by MOI and compared only with mutants in their bin. To provide a basis for normalizing between plates, wild-type *Mtb* control wells with MOI varying from 0.25 to 4 were included in each plate. Following addition of the bacteria to the macrophages, phagocytosis was allowed to proceed for four hours. Cells were then washed, and media was added back. After three days of infection, cells were again washed to remove extracellular bacteria, fixed, stained with A-R and DAPI, and imaged (**Fig. 1**).

**Image analysis: training set**

High-content imaging coupled with automated image analysis has the potential to quantify a variety of phenotypes relevant for cellular *Mtb* infection. We have used the approach in a small molecule screen to quantify *Mtb* fluorescence per macrophage [[1](#_ENREF_1)], and others have quantified early phagosome-lysosome fusion [[5](#_ENREF_5)]. However, other aspects of *Mtb* intracellular biology, including macrophage survival or death following infection, can similarly be quantitated. For this initial analysis, we focused on identifying mutants impaired for intracellular growth as defined by comparison with colony-forming units (CFU), the bacteriologic gold standard. As positive controls for this training set of images, we treated wild-type *Mtb*-infected macrophages with antibiotics to mimic a range of intracellular growth inhibition (**S1 Fig**). After imaging, we measured 616 readouts per macrophage, including morphology, intensity, and textural features of macrophage nuclei and stained *Mtb*. The full list of captured features has been described in a previous CellProfiler publication [[6](#_ENREF_6)]. Although in theory the full set of 616 metrics could be leveraged to build a machine-learning classifier that predicts CFU, we wanted to avoid over-training the system to the particularities of any specific positive control. Instead of using all 616 features, we thus chose initially to focus on three readily-interpretable features that correlated well with CFU: percent of total macrophages infected with *Mtb* (“Percent Infected”), normalized and integrated A-R fluorescent intensity (“*Mtb* fluorescence intensity”), and macrophage cell count (“Macrophage count,” given as a negative value) (**S1A Fig**). These metrics in fact capture several aspects of *Mtb* biology in macrophage cell culture. *Mtb* fluorescence intensity directly reflects intracellular bacterial burden, and reflects bacterial growth. In contrast, percent of macrophages infected and inverse macrophage cell count reflect *Mtb* effects on infected cells. Because virulent *Mtb* causes some degree of macrophage necrosis and subsequent bacterial release [[2](#_ENREF_2)], fully virulent bacteria are likely to induce more necrosis with subsequent infection of surrounding cells, resulting in a higher percentage of infection among remaining macrophages and fewer remaining macrophages overall. Thus integrated fluorescent intensity and percent of macrophages infected are higher and cell count is lower following infection with fully virulent *Mtb*. All of these metrics were independently identified to correlate with bacterial growth in a previous small molecule screen [[7](#_ENREF_7)].

**Pilot screen**

After optimizing the assay and determining the image features to measure, we performed a pilot screen on a single plate of mutants to assess assay performance. Each library plate was grown and prepared in duplicate; each duplicate was then used to infect two plates of macrophages, resulting in four replicates of each plate imaged. Mutant wells that failed to grow prior to infections were excluded from further consideration. After selection based on the three chosen imaging parameters, six hit mutants were retested by plating for CFU. Five of the six hit mutants were confirmed to be growth-impaired relative to control (**S1B Fig**). Principal component analysis (PCA) was then performed on the pilot data to determine whether a combination of measurements would best identify hits. Comparing hits based on the first principal component (PC1) with those called based on each individual feature, we determined that the PC1 was best able to distinguish true positives from false positives (**S1C Fig**). To test whether our PCA incorporating our three selected features or a PCA composed of all 616 measured imaging features would better predict mutants with impaired intracellular growth, we compared the performance of the two analyses for the pilot screen data (**S1D Fig**). Our three-feature PCA in fact performed better than the PCA incorporating all 616 features; this finding confirmed our concern that a model incorporating all 616 features might be overtrained to the training set of images. We thus elected to proceed with the screen using the three-feature PCA for mutant analysis.

**Gene-specific primers**

Cloning

*Hly* F gtagaaggaagtaaacccatga R ttattcgattggattatctactttattactatatttcggat

*drrC* F atgatcacgacgacaagtcaggaaa R tcaatgcgtgctggccc

*Rv0712* F gtgctgaccgagttggttgac R ctacccggacaccgggtc

*hrp1* F atgaccaccgcacgcga R ctagctggcgagggccatg

*serB1* F atggggctgacatgttggc R tcagcgtgattggcgtctagag

*Rv0771* F atgatggacgagctgcgcc R ctagctcgatttaccagattccttagc

PCR

*hly* PCR confirmation primers set 1 F acgcggatgaaatcgataag R cgcttttacgagagcacct

*hly* PCR confirmation primers set 2 F gcggatgaaatcgataagt R tcgcttttacgagagcacct

**Collision-induced dissociation major fragments for PDIM species**

Positive mode MS-MS

Parent m/z 1385.42 CID dominant fragments: 957.98, 929.95, 897.93, 487.52, 459.49

Parent m/z 1455.50 CID dominant fragments: 1001.03, 957.98, 925.95, 519.54, 487.52

Parent m/z 1411.44 CID dominant fragments: 957.98, 925.96, 487.52

Parent m/z 1467.50 CID dominant fragments: 972.00, 939.97, 487.52, 459.49

**Supplementary References.**

1. Stanley SA, Barczak AK, Silvis MR, Luo SS, Sogi K, et al. (2014) Identification of host-targeted small molecules that restrict intracellular Mycobacterium tuberculosis growth. PLoS Pathog 10: e1003946.

2. Behar SM, Divangahi M, Remold HG (2010) Evasion of innate immunity by Mycobacterium tuberculosis: is death an exit strategy? Nat Rev Microbiol 8: 668-674.

3. Keane J, Balcewicz-Sablinska MK, Remold HG, Chupp GL, Meek BB, et al. (1997) Infection by Mycobacterium tuberculosis promotes human alveolar macrophage apoptosis. Infect Immun 65: 298-304.

4. Lee J, Remold HG, Ieong MH, Kornfeld H (2006) Macrophage apoptosis in response to high intracellular burden of Mycobacterium tuberculosis is mediated by a novel caspase-independent pathway. J Immunol 176: 4267-4274.

5. Brodin P, Poquet Y, Levillain F, Peguillet I, Larrouy-Maumus G, et al. (2010) High content phenotypic cell-based visual screen identifies Mycobacterium tuberculosis acyltrehalose-containing glycolipids involved in phagosome remodeling. PLoS Pathog 6: e1001100.

6. Gustafsdottir SM, Ljosa V, Sokolnicki KL, Anthony Wilson J, Walpita D, et al. (2013) Multiplex cytological profiling assay to measure diverse cellular states. PLoS One 8: e80999.

7. Christophe T, Jackson M, Jeon HK, Fenistein D, Contreras-Dominguez M, et al. (2009) High content screening identifies decaprenyl-phosphoribose 2' epimerase as a target for intracellular antimycobacterial inhibitors. PLoS Pathog 5: e1000645.
