# Supplementary figures and images for: Systematic, multiparametric analysis of Mycobacterium tuberculosis intracellular infection offers insight into coordinated virulence
Source: PLoS Pathog. 2017 May 15;13(5):e1006363. doi: 10.1371/journal.ppat.1006363 (PMC5444860; doi:10.1371/journal.ppat.1006363)

S1 Fig

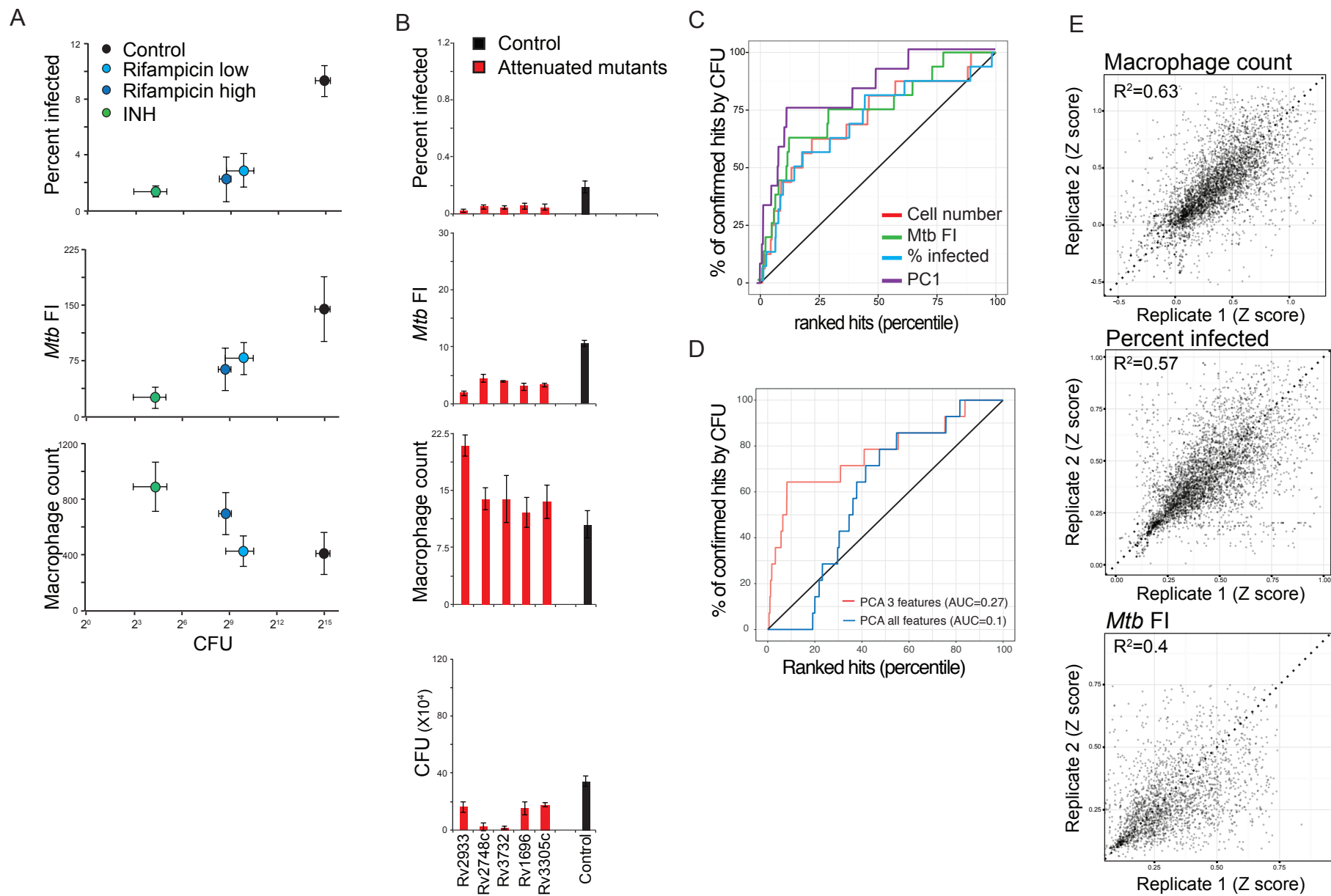

Supplement: S1 Fig — (A) Mtb strain H37Rv was used to infect J774A.1 cells simultaneously for imaging and for colony-forming units (CFU) determination. Cells were treated with isoniazid (1μg/ml) or rifampicin (0.025μM or 0.25μM). (B) Comparison of individual imaging outputs and CFU for six growth-impaired mutants identified in the pilot screen. Five out of six were confirmed attenuated by CFU (data shown for the five attenuated mutants) (C) Transposon mutants were ranked (x-axis) by one of four metrics: percent macrophages infected, Mtb fluorescence intensity (Mtb FI), macrophage cell count, or a primary component analysis (PCA) incorporating all three metrics, against percentage of mutants confirmed to have impaired intracellular growth by CFU (y-axis). PCA best distinguished true positives. (D) Transposon mutants were ranked (x-axis) by their score in the three-feature PCA (“PCA 3 features”) or a PCA incorporating data for all 616 imaging features against the percentage of mutants confirmed by CFU (y-axis). The three-feature PCA better distinguished true positives. (E) Replicate reproducibility of the three individual imaging metrics for the full screen. (PDF) [file ppat.1006363.s002.pdf]

### Screening strategy

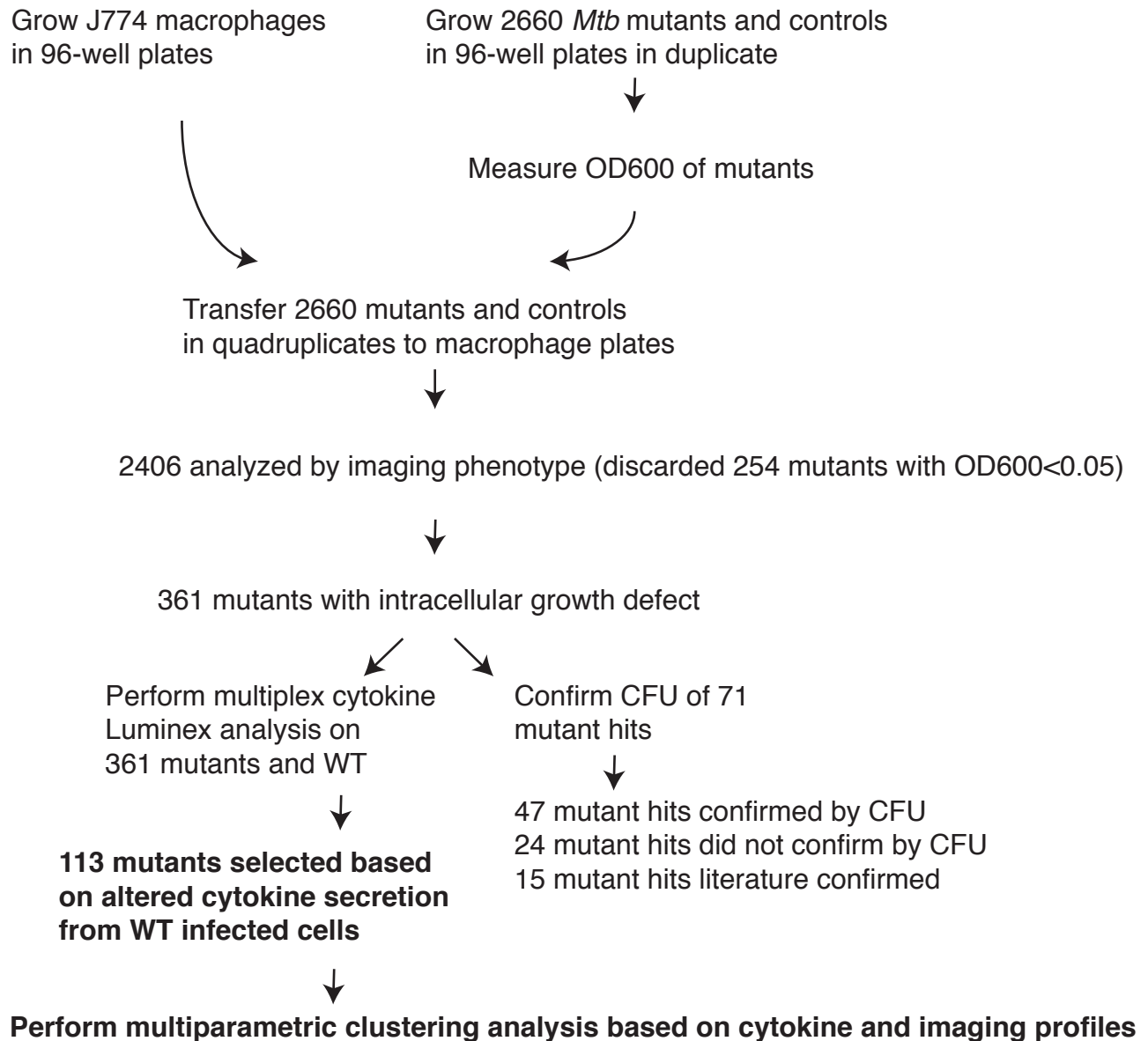

Supplement: S2 Fig — (PDF) [file ppat.1006363.s003.pdf]

S3 Fig

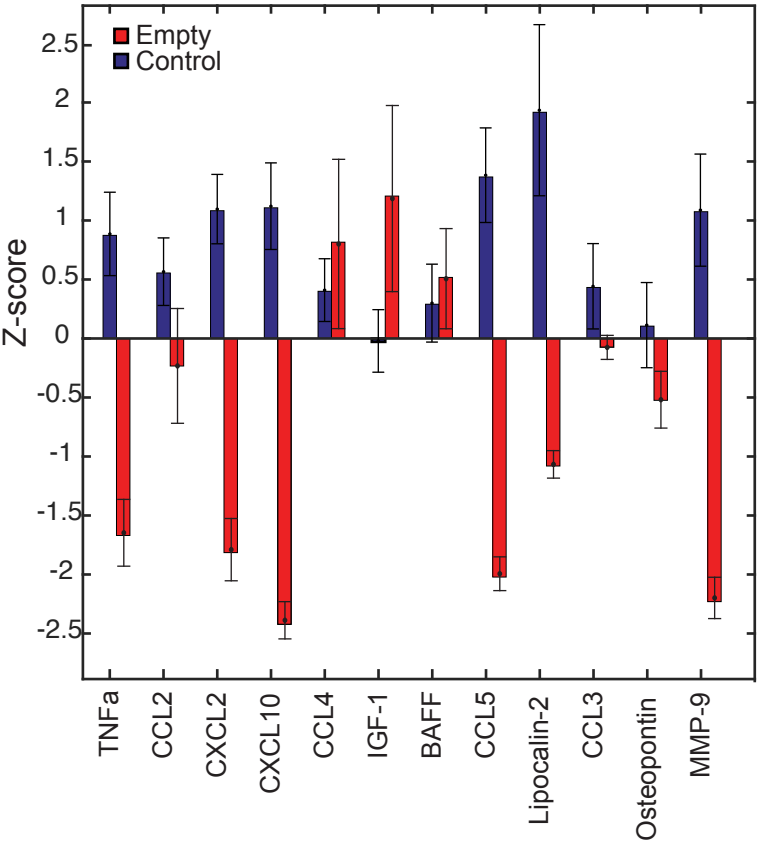

Supplement: S3 Fig — To determine whether the dynamic range for each measured cytokine was broad enough to allow meaningful interpretation of mutant results falling between wild-type infected cells and uninfected cells, we compared the normalized cytokine values for wild-type-infected and uninfected cells. Shown are normalized Z-scores of detection of the indicated cytokines of uninfected (red) and control-infected (blue) cells. Values represent +/- SD for three independent replicates. (PDF) [file ppat.1006363.s004.pdf]

S4 Fig

A

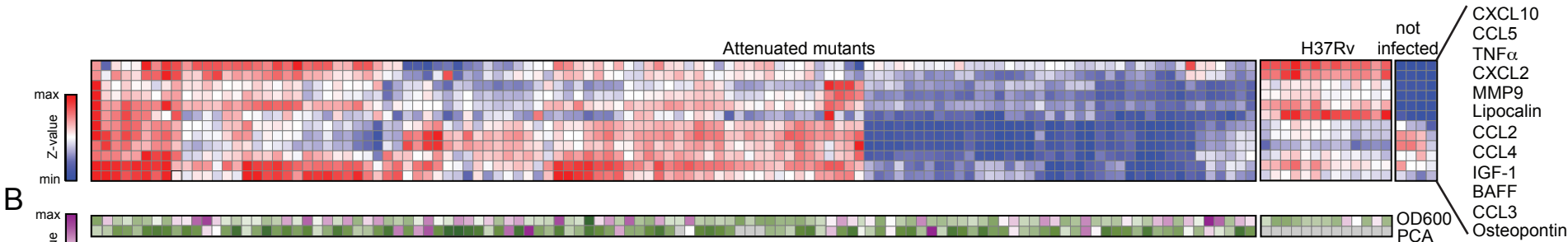

B

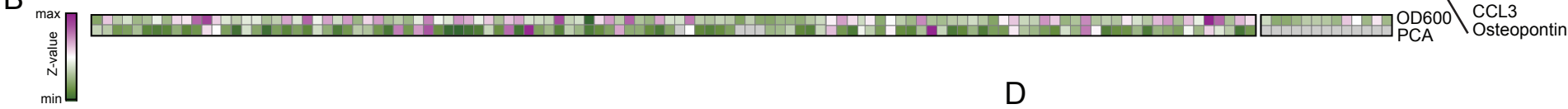

C

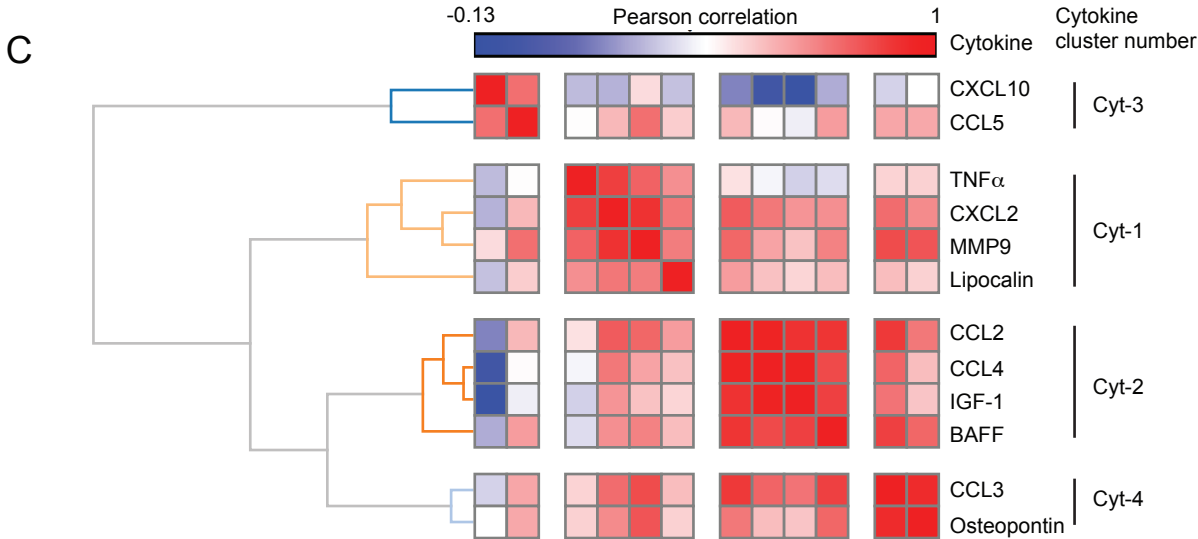

D

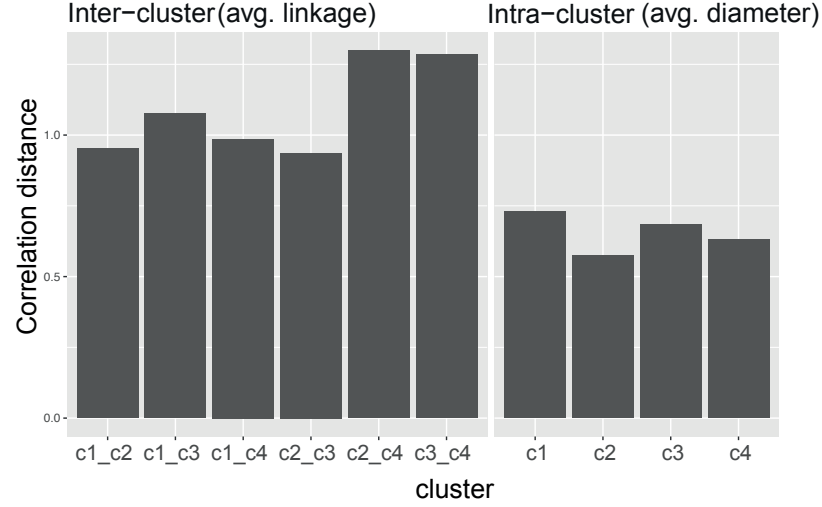

Supplement: S4 Fig — (A) Macrophages were infected with the 361 individual mutants. Supernatants were collected after 3 days of infection and cytokines were measured by Luminex multiplexed cytokine quantitation. The heatmap (red-blue) shows the 113 mutants inducing at least a two-fold difference in two cytokines from H37Rv wild-type-infected macrophages. (B) The input OD600 and image analysis PC1 (purple-green) for each of the 113 mutants plotted below shows that the cytokine clustering does not correlate with either parameter. (C) Hierarchical clustering of the 12 cytokines into 4 groups based on Pearson correlation for behavior across all hit mutants. (D) correlation distance for inter-cluster and intra-cluster comparisons. In all cases, intra-cluster comparisons have a smaller correlation distance than inter-cluster comparisons. (PDF) [file ppat.1006363.s005.pdf]

S5 Fig

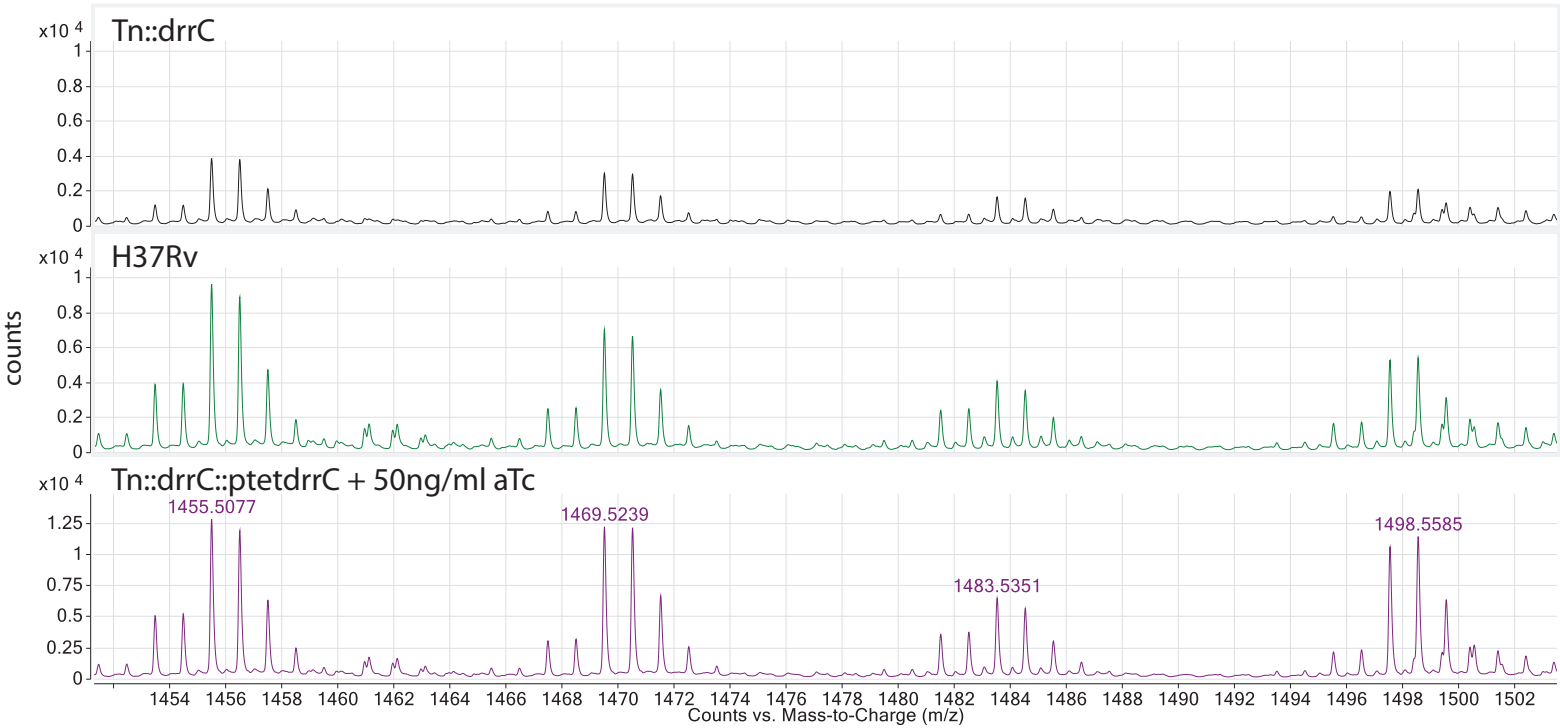

Supplement: S5 Fig — Bacteria were grown to late log phase in 7H9 with OADC, glycerol, and tween 0.05%. Cells were then pelleted, and supernatant was harvested, sterile filtered, and extracted with hexanes. Hexane extracts were then extracted with water 5 times to reduce background tween contamination. LC-MS was then performed on the extracts according to established protocols [48]. Raw mass spectra were extracted over the retention time where PDIM species (as confirmed by collision-induced dissociation fragmentation patterns) ran. (PDF) [file ppat.1006363.s006.pdf]

S6 Fig

A

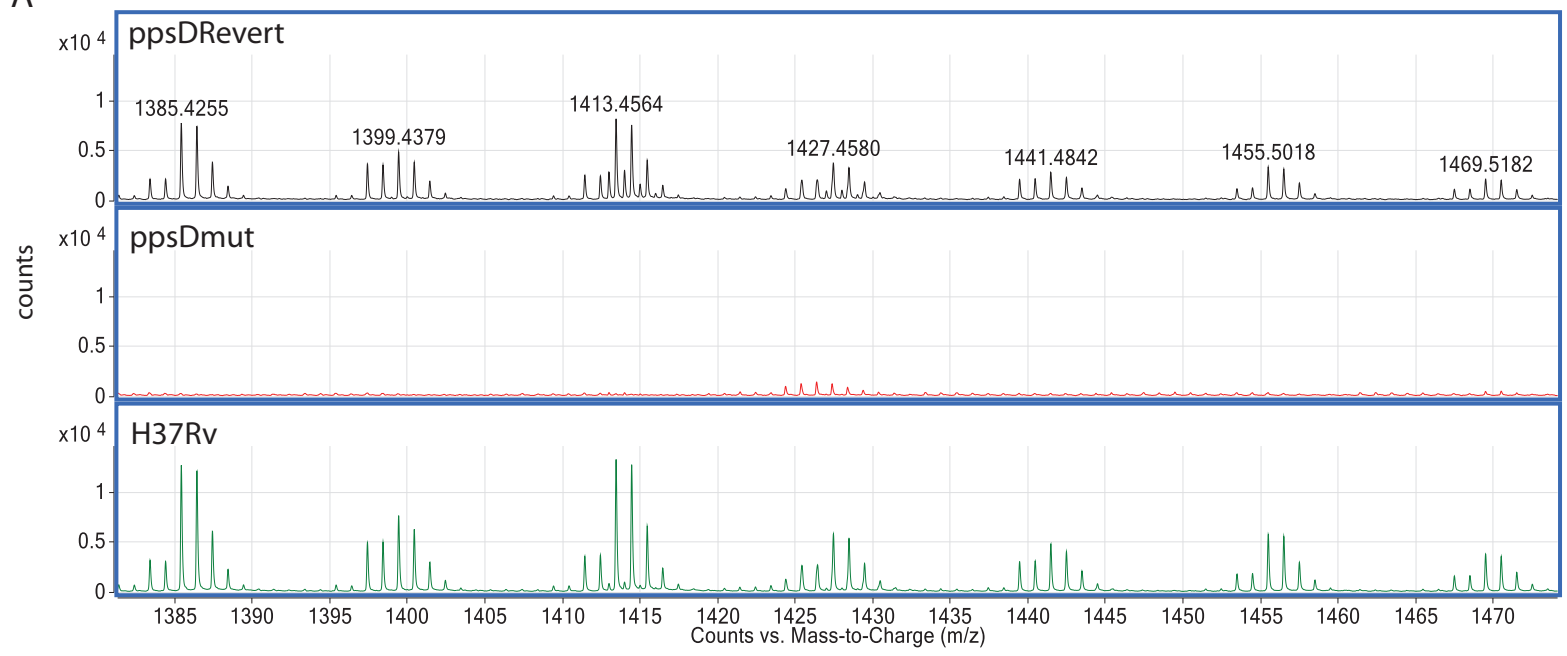

B

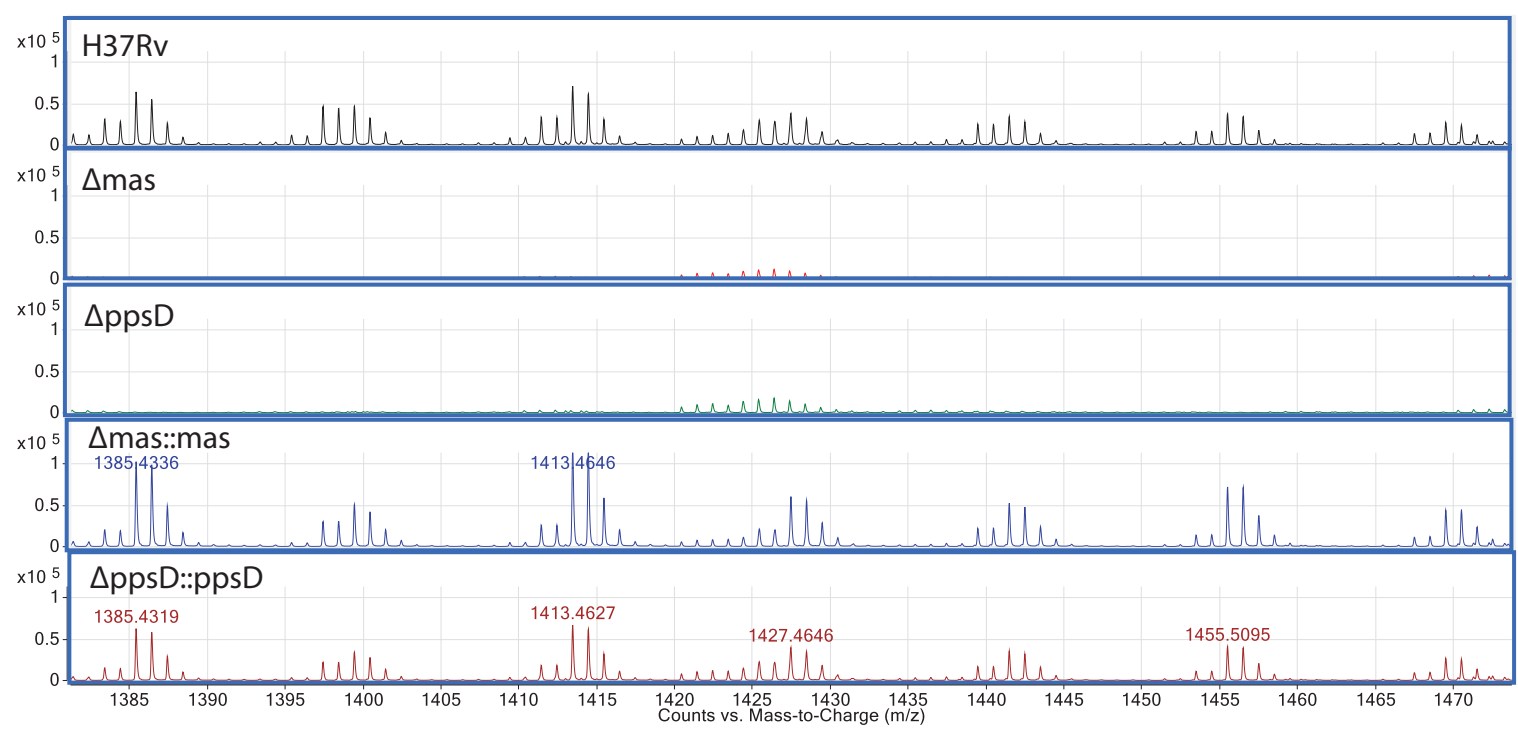

Supplement: S6 Fig — Bacteria were grown to OD600 0.6 +/- 0.1 in media lacking tween. Cells were then pelleted, and total cell-wall lipids were extracted in chloroform:methanol. LC-MS was performed on the extracts according to established protocols [48]. Raw mass spectra were extracted over the retention time where PDIM species (as confirmed by collision-induced dissociation fragmentation patterns) ran. A. Spectra for the H37Rv and the ppsD point mutant strain (ppsD(G44C)) and chromosomal reversion. B. Spectra for H37Rv and the ppsD and mas clean deletions and complements. (PDF) [file ppat.1006363.s007.pdf]

S7 Fig

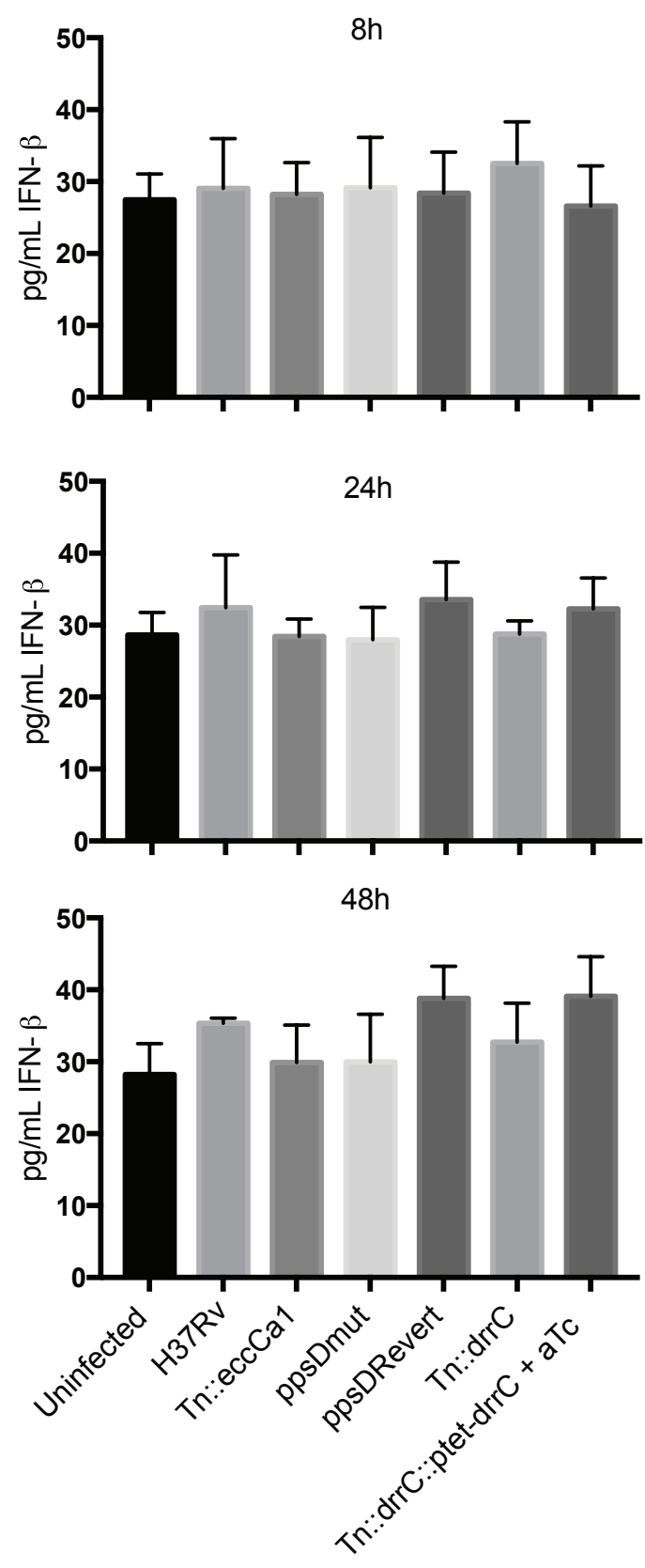

Supplement: S7 Fig — Bone marrow-derived murine macrophages were infected with the indicated strains at an MOI of 2:1. After a 4 hour phagocytosis step, cells were washed to remove remaining extracellular bacteria and media was added back. Supernatants were harvested for ELISAs at the indicated times after the initiation of infection. Mean +/- SD for 3 replicates. (PDF) [file ppat.1006363.s008.pdf]

S8 Fig

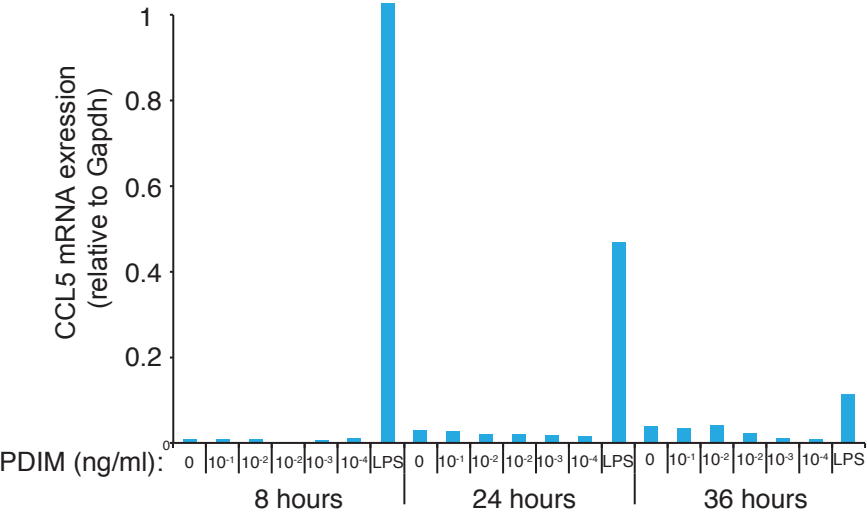

Supplement: S8 Fig — Bone marrow-derived macrophages were stimulated with either LPS (100ng/ml) or with the indicated doses of purified PDIM. Cells were harvested at the indicated time points for RNA extraction and cDNA preparation. Real-time qPCR was then used to quantitate expression of type I IFN-responsive gene CCL5. Gapdh was used as a normalizing control for each sample. (PDF) [file ppat.1006363.s009.pdf]

S9 Fig

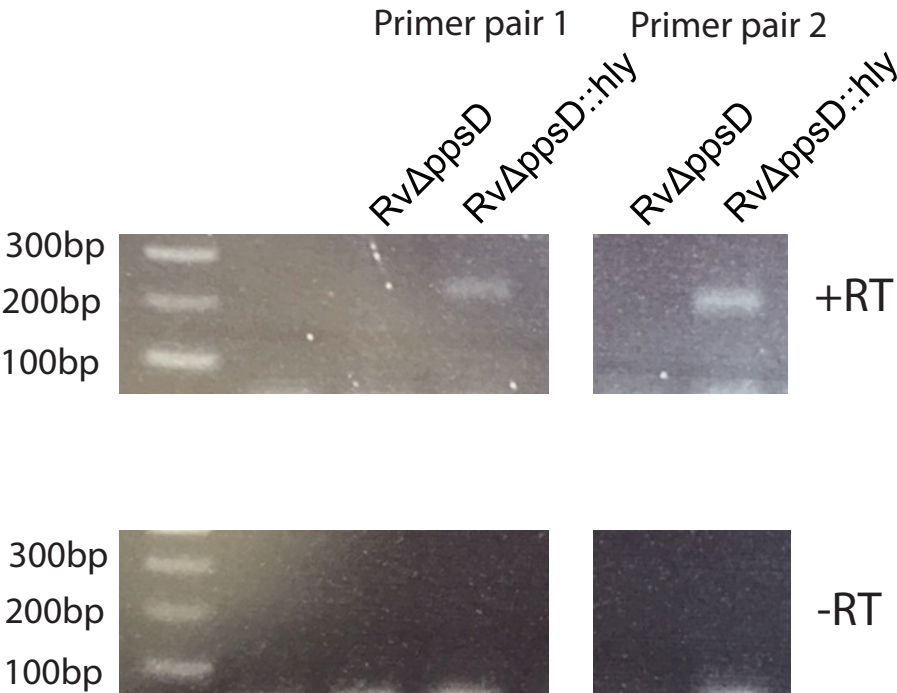

Supplement: S9 Fig — Macrophages were infected with ΔppsD or ΔppsD::hly at an MOI of 2:1. Mixed macrophage and bacterial RNA was harvested 24 hours after infection for cDNA preparation. PCR with primers specific to hly was then performed. Hly transcript was detectable from cDNA prepared with reverse transcriptase (+RT) but not from the control sample without reverse transcriptase added (-RT). (PDF) [file ppat.1006363.s010.pdf]

S10 Fig

Supernatants

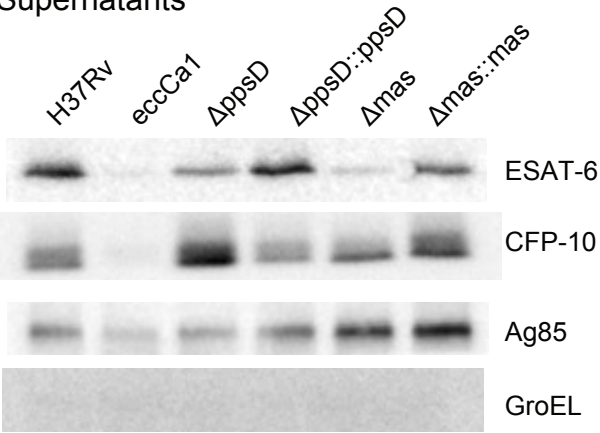

Pellets

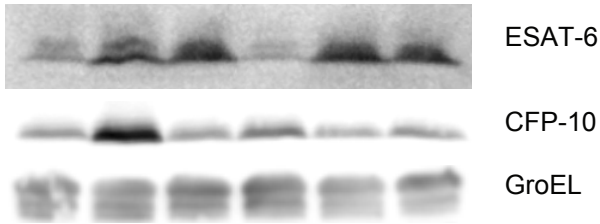

Supplement: S10 Fig — Supernatants from wild-type, ESX-1 mutant (eccCa1), or PDIM mutants and complements were harvested, concentrated, and probed for ESAT-6 or CFP-10, antigen 85 (Sec-secreted control), and GroEL (lysis control). Corresponding pellets were simultaneously lysed and probed for ESAT- or CFP-10 (production control) and GroEL. Disruption of mas results in loss of ESAT-6 and CFP-10 secretion; restoring mas on a constitutively expressing episomal plasmid restores the wild-type phenotype. The ppsD clean deletion mutant shares the phenotype of the ppsD point mutant (ppsD(G44C)) shown in Fig 4; ESAT-6 secretion is diminished and CFP-10 secretion is enhanced. Complementation with an episomal plasmid constitutively expressing the operon including ppsD restores the wild-type phenotype. (PDF) [file ppat.1006363.s011.pdf]

S11 Fig

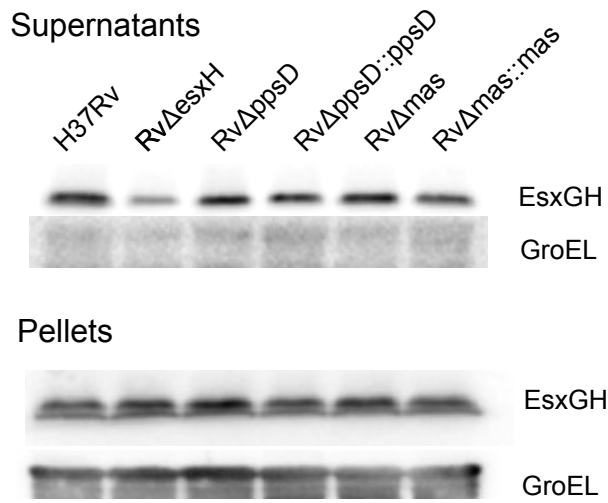

Supplement: S11 Fig — Bacteria were grown to mid-log phase, then bacteria were washed twice in Sauton’s medium that had been pre-chelated. After 48 hours of growth, cells were pelleted and supernatants were harvested, concentrated, and run on a Tris-glycine gel. Blots were probed with antibody to EsxGH (gift of Dr. Jennifer Philips, Washington University), and GroEL (lysis control). Corresponding pellets were simultaneously probed for EsxGH and GroEL. (PDF) [file ppat.1006363.s012.pdf]

S12 Fig

A

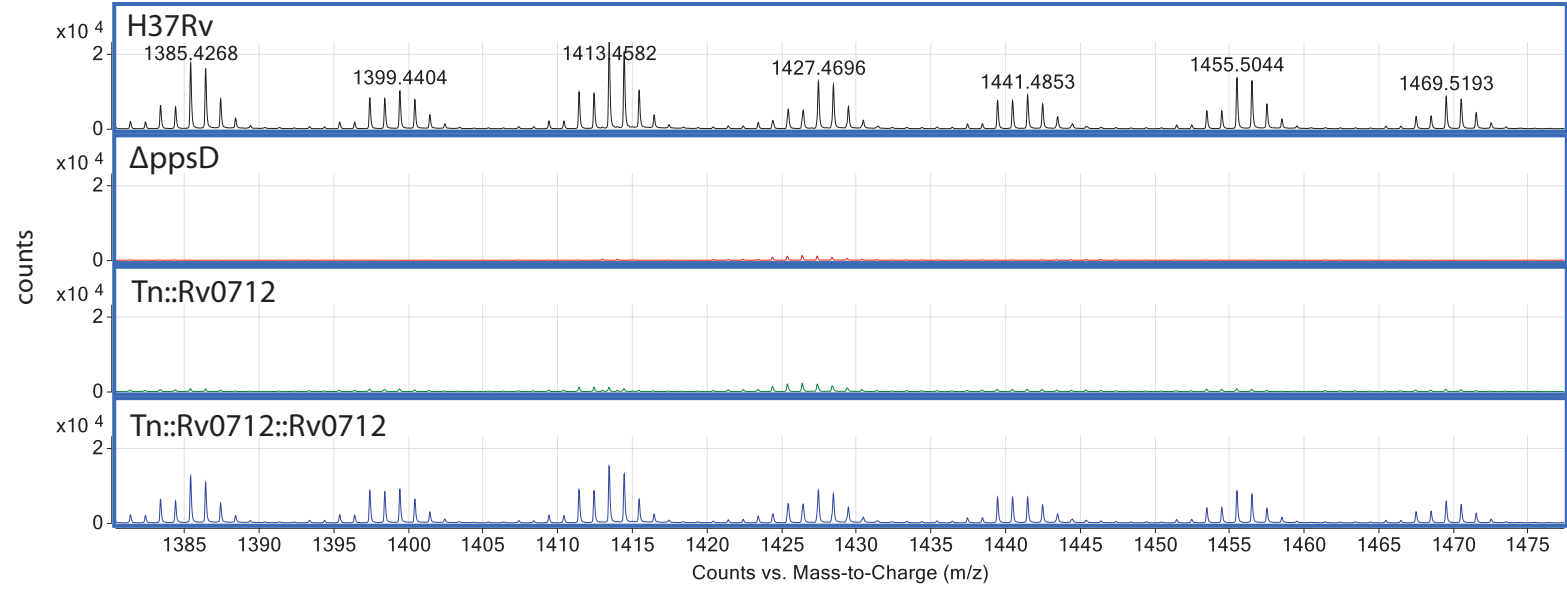

B

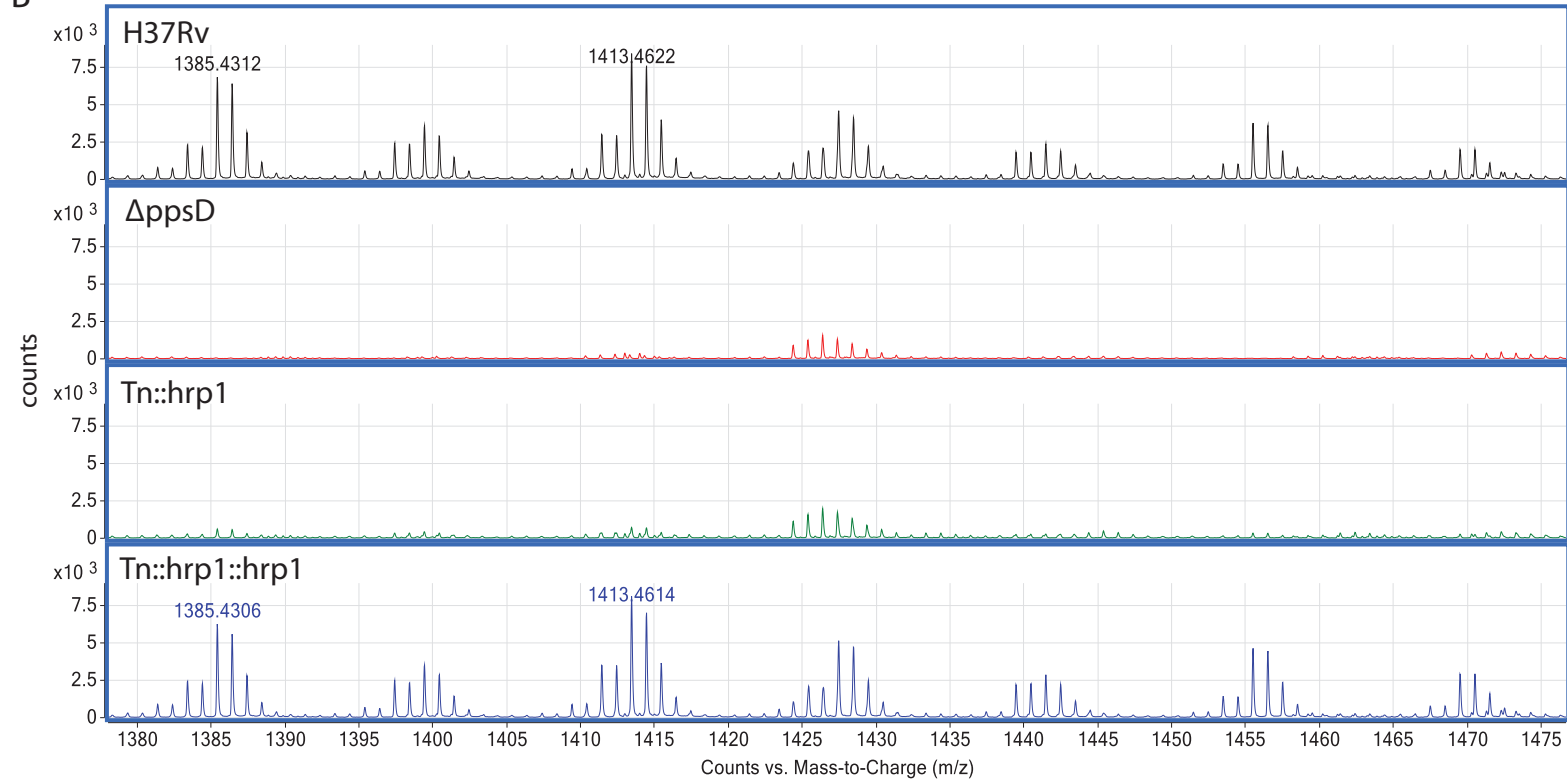

Supplement: S12 Fig — Bacteria were grown to OD600 0.6 +/- 0.1 in media lacking tween. Cells were then pelleted, and total cell-wall lipids were extracted in chloroform:methanol. LC-MS was performed on the extracts according to established protocols [48]. Raw mass spectra were extracted over the retention time where PDIM species (as confirmed by collision-induced dissociation fragmentation patterns) ran. A. Raw spectra for H37Rv, the ppsD clean deletion (PDIM negative control), Tn::Rv0712 and Tn::Rv0712 complemented with an episomal plasmid constitutively expressing the gene. B. Spectra for H37Rv, the ppsD clean deletion (PDIM negative control), Tn::hrp1, and Tn::hrp1 complemented with an episomal plasmid constitutively expressing the gene. (PDF) [file ppat.1006363.s013.pdf]

S13 Fig

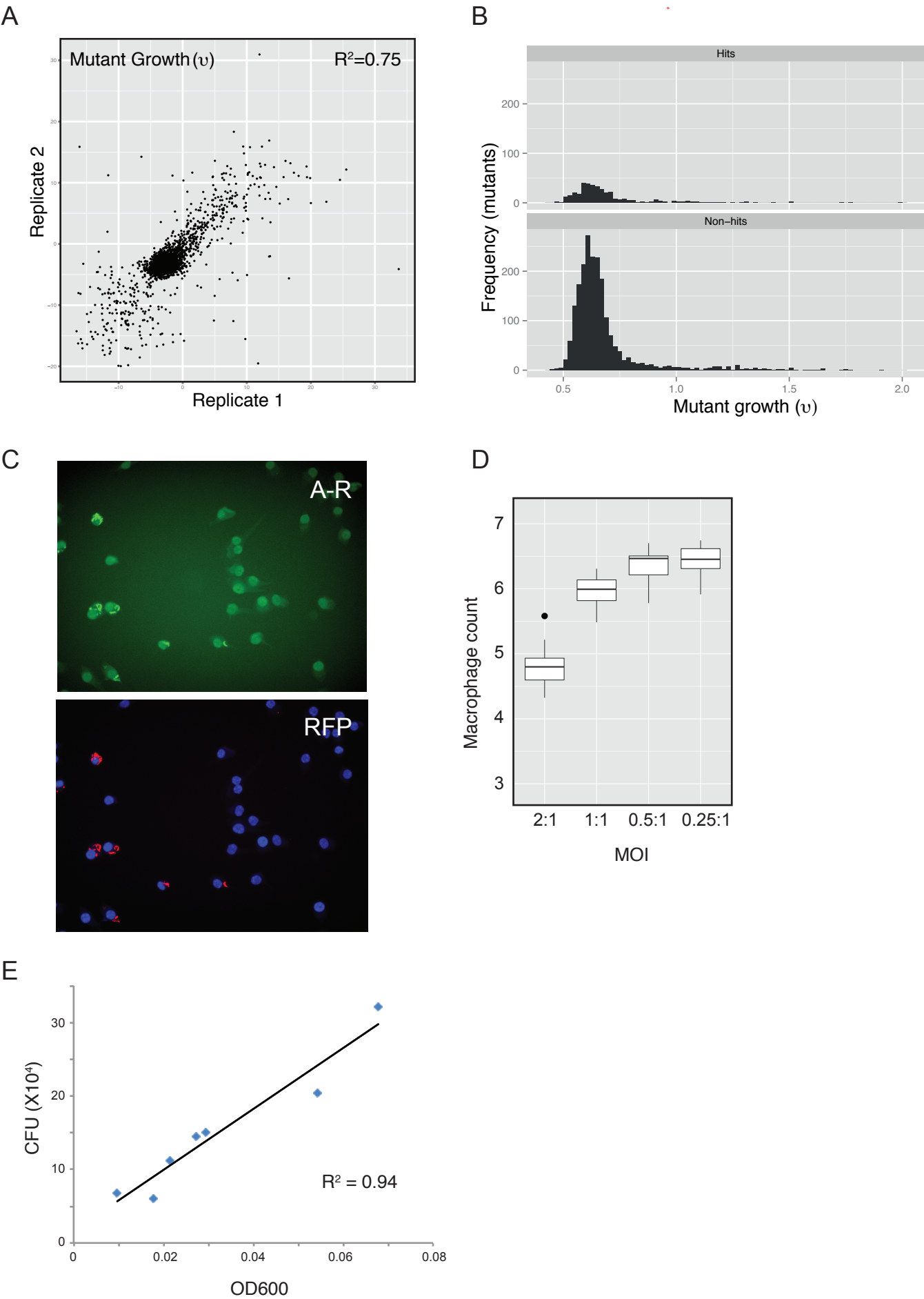

Supplement: S13 Fig — The Mtb transposon mutant library was grown in liquid culture for 14 days. Growth was assessed every 2–3 days by OD600 after mixing. A ν value for growth in liquid culture was then calculated for each mutant. (A) Biological replicate correlation. (B) Histogram of ν values for screen hits or non-hits. The calculated value for each mutant represents the mean of two independent replicates. Screen hits and non-hits have similar growth rates in liquid culture. (C-D) Wild-type Mtb strains H37Rv or H37Rv expressing RFP were grown to the shown densities, washed in PBS, and used to infect J774A.1. After a 4 hour phagocytosis, cells were washed to remove any unengulfed Mtb. (C) H37Rv::RFP infected cells were stained with auramine-rhodamine (A-R). Red and green fluorescence were compared to determine how well A-R staining identified Mtb. (D) Cells infected at the given MOIs were incubated for 3 days. Cells were then washed, fixed, and macrophage nuclei were stained with DAPI. Plates were then imaged and nuclei were quantitated using automated image analysis. Two-fold differences in MOI significantly change macrophage survival. (E) Following infection with H37Rv, macrophages were lysed and plated for CFU. Input OD600 was found to correlate well with quantitation of internalized Mtb. (PDF) [file ppat.1006363.s014.pdf]
